# Structural characterization of the ABC transporter DppABCDF in *Escherichia coli* reveals insights into dipeptide acquisition

**DOI:** 10.1371/journal.pbio.3003026

**Published:** 2025-03-07

**Authors:** Panpan Li, Manfeng Zhang, Yihua Huang

**Affiliations:** 1 National Laboratory of Biomacromolecules, CAS Center for Excellence in Biomacromolecules, Institute of Biophysics, Chinese Academy of Sciences, Beijing, China; 2 University of Chinese Academy of Sciences, Beijing, China; Rutgers University-Robert Wood Johnson Medical School, UNITED STATES OF AMERICA

## Abstract

The prokaryote-specific ATP-binding cassette (ABC) peptide transporters are involved in various physiological processes and plays an important role in transporting naturally occurring antibiotics across the membrane to their intracellular targets. The dipeptide transporter DppABCDF in Gram-negative bacteria is composed of five distinct subunits, yet its assembly and underlying peptide import mechanism remain elusive. Here, we report the cryo-EM structures of the DppBCDF translocator from *Escherichia coli* in both its apo form and in complexes bound to nonhydrolyzable or slowly hydrolyzable ATP analogs (AMPPNP and ATPγS), as well as the ATPγS-bound DppABCDF full transporter. Unlike the reported heterotrimeric *Mycobacterium tuberculosis* DppBCD translocator, the *E. coli* DppBCDF translocator is a heterotetramer, with a [4Fe-4S] cluster at the C-terminus of each ATPase subunit. Structural studies reveal that ATPγS/AMPPNP-bound DppBCDF adopts an inward-facing conformation, similar to that of apo-DppBCDF, with only one ATPγS or AMPPNP molecule bound to DppF. By contrast, ATPγS-bound DppABCDF adopts an outward-facing conformation, with two ATPγS molecules glueing DppD and DppF at the interface. Consistent with structural observations, ATPase activity assays show that the DppBCDF translocator itself is inactive and its activation requires concurrent binding of DppA and ATP. In addition, bacterial complementation experiments imply that a unique periplasmic scoop motif in DppB may play important roles in ensuring dipeptide substrates import across the membrane, presumably by preventing dipeptide back-and-forth binding to DppA and avoiding dipeptides escaping into the periplasm upon being released from DppA.

## Abstract 2

Bacterial ABC peptide transporters are involved in important physiological processes, such as nutrient uptake. This study solves the cryo-EM structures of the dipeptide transporter DppABCDF from E. coli in both its apo form and bound to nucleotide analogs, providing insights into its assembly and underlying peptide import mechanism.

## Introduction

The prokaryote-specific ATP-binding cassette (ABC) peptide transporters, a subfamily of type I ABC importers, are involved in various physiological processes such as nutrient uptake [1], cell-wall peptides recycling [2], sporulation [3] and host immune regulation [4]. This family of importers also plays an important role in transporting naturally occurring antibiotics, such as negamycin [5], pacidamycin [6] and others [7–12], across the membrane to their intracellular targets. Different from those structurally and mechanistically well-characterized ABC exporters that solely consist of two variable transmembrane domains (TMDs) and two highly conserved nucleotide-binding domains (NBDs), the ABC peptide importers require an additional substrate-binding protein (SBP) that captures peptide substrates and delivers them to the translocator for function [13–17]. Structurally, each SBP is composed of an N-terminal lobe (N-lobe) and a C-terminal lobe (C-lobe), connected by a hinge region (S1 Fig) [18–22]. It is generally believed that SBPs employ a “Venus flytrap” mechanism for substrate capture, adopting an open, substrate-accessible conformation in the absence of substrate and a clamped conformation upon substrate binding (S1 Fig) [18–22]. The peptide-binding SBPs belong to the cluster C SBPs, which have a larger cleft between two lobes, providing more space to accommodate relatively larger ligands, such as oligopeptides [23,24].

*Escherichia coli* possess two prototypes of ABC peptide transporters or permeases: the Dpp transporter (DppABCDF) and the Opp transporter (OppABCDF) that intake peptides with length ranging from 2 to 5 amino acid residues from the periplasmic space [25,26]. The Dpp transporter is primarily responsible for importing dipeptides but also capable of inefficiently importing tri- and tetrapeptides into the cell [25,26]. In Gram-negative bacteria, the full Dpp transporter is composed of five subunits: two integral membrane proteins (DppB and DppC) that form a translocation pathway for substrates; two ATPases (DppD and DppF) that bind and hydrolyze ATP during transport and a periplasm-localized SBP (DppA) for substrate capture. While it is generally believed that SBP proteins work in synergy only with their cognate translocators in the transport process [26], MppA, an isolated cluster C SBP in *E. coli* that shares 46% overall amino acid sequence identity with OppA and 29% identity with DppA, is able to facilitate uptake of murein tripeptide (l-Ala-γ-d-Glu-meso-Dap, Mtp) by utilizing Opp translocator for peptidoglycan biosynthesis [26,27], and Dpp translocator for heme acquisition [28–30].

Recent structural studies greatly advance our understanding of the mechanisms underlying substrate recognition and transport for ABC peptide transporters. Hu and colleagues reported cryo-EM structures of DppABCD from *M. tuberculosis (Mtb)* in different conformational states, providing a molecular roadmap for understanding the transport mechanism of a cluster C SBP and its translocator [17]; they further determined the cryo-EM structures of *Mtb*OppABCD, disclosing the importance of an iron-sulfur cluster-bound domain within *Mtb*OppD in the assembly of the transporter [16]. However, the composition and assembly of ABC peptide transporters are highly diversified in different bacterial strains. For instance, the SBPs are membrane-anchored lipoproteins in *M. tuberculosis* and other Gram-positive bacteria [31,32], yet their counterparts are soluble periplasmic proteins in Gram-negative bacteria. In addition, in both *Mtb*OppABCD and *Mtb*DppABCD, their translocators are heterotrimeric complexes in which DppD and OppD each contains two consecutive NBDs, connected with or without an iron-sulfur cluster-bound domain [16,17]. By contrast, the translocators DppBCDF and OppBCDF in Gram-negative bacteria are heterotetrameric complexes in which the two copies of NBDs are encoded by two different genes (*dppD* and *dppF* in Dpp or *oppD* and *oppF* in Opp).

To elucidate the molecular mechanisms of the peptide transporters in Gram-negative bacteria, we determined cryo-EM structures of the Dpp translocator DppBCDF from *E. coli* both in apo- and ATPγS or AMPPNP-bound forms, as well as the ATPγS-bound DppABCDF full transporter. Structural studies reveal that DppBCDF and ATP analog-bound DppBCDF adopt a similar inward-facing conformation with one ATPγS or AMPPNP molecule bound to its one NBD domain, DppF. Corroborating our structural observations, DppBCDF lacks ATPase activity and its activation requires the presence of DppA. In line with the ATPase activity assay results, cryo-EM structure of DppABCDF in complex with ATPγS reveals an outward-facing conformation with two ATPγS molecules glueing the two NBD domains, DppD and DppF, at the interface. Bacterial complementation experiments imply that a unique periplasmic scoop motif (α1-loop-α2 motif) in DppB may play important roles in ensuring dipeptide substrates import across the membrane, presumably by preventing dipeptide back-and-forth re-binding to DppA and averting dipeptides escaping into periplasm upon released from DppA.

## Results

### Overall structure of DppBCDF from *E. coli*

**Fig 1:**
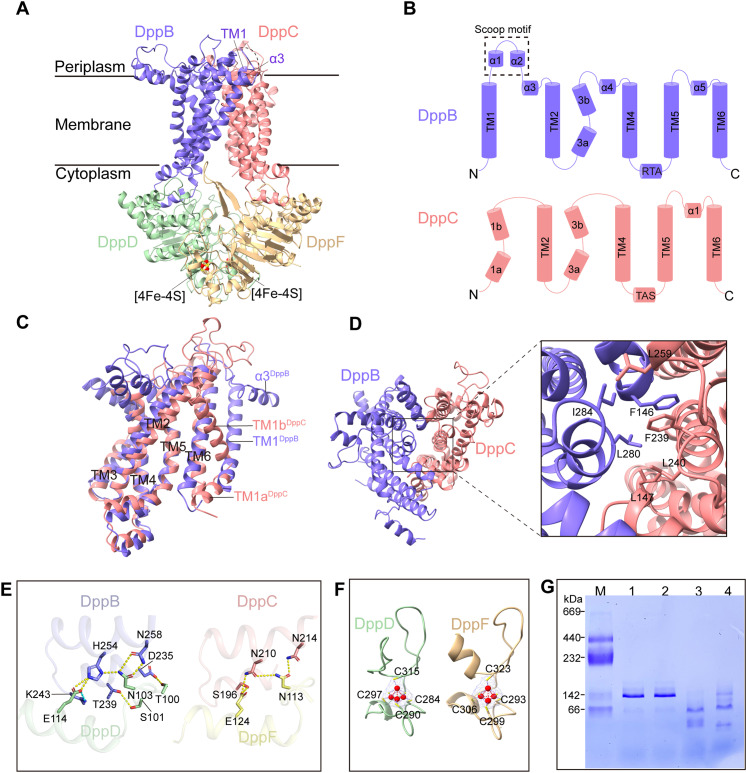
Cryo-EM structure of the DppBCDF translocator. (**A**) Cartoon representation of the DppBCDF structure. DppB, DppC, DppD and DppF are colored slate blue, light coral, sea green and burly wood, respectively. The invisible region in DppB (residues 32–62) is connected with a dotted line. Two [4Fe-4S] clusters are labeled. (**B**) Topological diagram of DppB (slate blue) and DppC (light coral). The scoop motif connecting TM1 with α3 helices in DppB is invisible in the DppBCDF structure. The two coupling helices (also known as EAA loops) are labeled as “RTA” in DppB and “TAS” in DppC according to the sequence. (**C**) Superposition of DppB (slate blue) and DppC (light coral) structures showing the differences in conformation of TM1 and composition of TM1-TM2 and TM3-TM4 periplasmic regions. (**D**) Close-up view of the periplasmic substrate entrance of the substrate translocation pathway. Resides F164, I284 and L280 of DppB form extensive hydrophobic interactions with residues L147, F239, L240 and L259 of DppC. (**E**) Close-up view of DppB–DppD interactions (left) and DppC–DppF interactions (right). (**F**) The iron-sulfur cluster-bound domain in DppD (left) and DppF (right). The [4Fe-4S] cluster in DppD is coordinated by Cys284, Cys290, Cys297 and Cys315 in DppD; the [4Fe-4S] cluster in DppF is coordinated by Cys293, Cys299, Cys306 and Cys323 in DppF. The [4Fe-4S] clusters are shown in ball-and-stick model, with iron atoms shown in yellow and sulfur atoms shown in red. (**G**) Blue Native-polyacrylamide gel electrophoresis (BN-PAGE) analysis of the DppBCDF complex in different buffer conditions. M, standard molecular mass marker; lane 1, purified DppBCDF complex; lane 2, DppBCDF in a buffer containing 2 mM DTT; lane 3, DppBCDF in a buffer containing 2 mM (NH_4_)_2_S_2_O_8_; lane 4, DppBCDF in a buffer containing 2 mM CuCl_2_. The data underlying this figure can be found in S1 Raw Images.

The *E. coli* gene cluster *dppABCDF* encoding a full Dpp transporter DppABCDF is sequentially organized in one operon (S2A Fig). To obtain the structure of the Dpp translocator DppBCDF, recombinant DppBCDF tetrameric complex was purified to homogeneity and prepared in a buffer containing 0.006% glyco-diosgenin (GDN) for sample freezing (S2B Fig). The DppBCDF structure was determined to a global resolution of 3.2 Å (S2C and S2F Fig). In the final model, except 30 residues, *i.e.*, the periplasmic “scoop motif” region that connects transmembrane (TM) helix TM1 with α3 short helix in DppB, other residues in the DppBCDF complex are unambiguously resolved (Fig 1A and 1B). The DppBCDF translocator assembles to form a rare heterotetramer with DppB and DppC specifically interacting with DppD and DppF, respectively, exhibiting a pseudo-dimeric symmetry perpendicular to the membrane plane (Fig 1A). The two integral membrane proteins, DppB and DppC, adopt a similar overall fold, each containing six TM helices, yet they deviate strikingly in conformation of TM1 and composition of TM1-TM2 and TM3-TM4 periplasmic regions (Figs 1B and 1C and S3A and S3B). In the complex, TM helices (TM3, TM4, TM5 and TM6) of both DppB and DppC converge to form a substrate translocation pathway, yet its periplasmic substrate entrance is occluded via hydrophobic interactions (Fig 1D). However, on the cytoplasmic side, TM helices (TM3, TM4, TM5 and TM6) of DppB are well separated apart from those of DppC, generating an inverted “V-shaped” substrate translocation pathway open to the cytoplasm (Fig 1A). In the tetrameric complex, each TMD–NBD interaction is mediated by a coupling helix (also referred as the EAA motif) that links TM4 with TM5 of each TMD in the cytoplasm [33,34] (Fig 1B). The DppB–DppD interactions are mediated by multiple inter-subunit hydrogen bonds and a K243^DppB^-E114^DppD^ salt bridge (Fig 1E, left), whereas the DppC–DppF interactions are predominantly inter-subunit hydrogen bonds (Fig 1E, right). Amino acid sequence alignments show that residues involved in each TMD–NBD interaction are well conserved in different bacterial homologs, arguing that the differential TMD–NBD interactions in DppBCDF may ensure the precise assembly of the heterotetrameric complex (S4A and S4B Fig). In line with the inward-facing conformation of the two TMDs, the two NBDs, DppD and DppF, also adopt an open conformation with no bound nucleotides observed at the interface (Fig 1A). Apparently, the current structure snapshots an inward-facing resting state of the DppBCDF translocator.

Notably, the two ATPases, DppD and DppF, in the complex, each binds a [4Fe-4S] cluster via their respective C-terminal domain (Fig 1A and 1F), consistent with the crystal structure of the isolated DppD from Gram-negative bacteria *Thermoanaerobacter tengcongensis* [35]. Previous mutagenesis studies show that the iron-sulfur cluster of *Mtb*OppD plays a critical role in stabilizing the *Mtb*OppABCD complex [16]. Consistent with these findings, Cys-to-Ser mutations of each iron-sulfur cluster-bound residues in either DppD or DppF caused disassembly of the DppBCDF complex (S5A Fig). Blue Native-polyacrylamide gel electrophoresis (BN-PAGE) analysis show that oxidation of the [4Fe-4S] by addition of oxidants such as CuCl_2_ and (NH_4_)_2_S_2_O_8_ also resulted in dissociation of the pre-formed complexes (Fig 1G). Indeed, if no reducing reagents such as dithiothreitol (DTT) or tris (2-carboxyethyl) phosphine hydrochloride (TCEP) were provided, the DppBCDF sample became strikingly morphologically heterogeneous as observed under cryo-EM (S5B Fig). Collectively, our findings show that each iron-sulfur cluster in DppBCDF is indispensable, reinforcing the notion that the iron-sulfur cluster play critical roles in stabilizing and assembling the peptide translocators. However, apart from stabilizing and assembling the translocator complex, other functions of the iron-sulfur cluster-bound domains in DppBCDF await further investigation.

A structural comparison between DppBCDF and the resting-state structures of *Mtb*DppABCD/*Mtb*OppABCD shows that DppBCDF aligns well with *Mtb*DppBCD and *Mtb*OppBCD, with root mean square deviations (RMSDs) of 1.69 Å (943 Cα atoms) and 1.38 Å (883 Cα atoms), respectively (S6 Fig). Notable differences are observed near the periplasmic-facing regions of the TM subunits, which may be related to their interactions with the respective SBPs. The iron-sulfur cluster-bound domain in *Mtb*OppD aligns well with that in DppD; however, *Mtb*DppD lacks any iron-sulfur cluster-bound domains, and the second iron-sulfur cluster-bound domain, found exclusively in DppF, is absent in *Mtb*OppD.

### ATP binding is insufficient to induce conformational changes of DppBCDF

**Fig 2:**
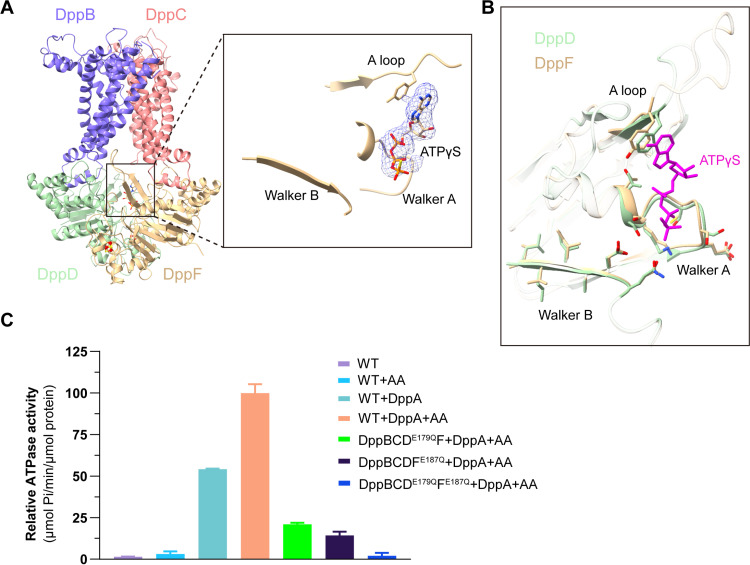
Cryo-EM structure of ATPγS-DppBCDF. (**A**) Cartoon representation of the ATPγS-DppBCDF structure. Color scheme same as in Fig 1. The EM density of ATPγS in DppF is shown on the right, and an ATPγS molecule is modeled in the EM density. (**B**) Structural overlay of two ATPases, DppD and DppF in ATPγS-DppBCDF showing their respective Walker A, Walker B and A loop motifs. ATPγS bound with DppF is colored violet. (**C**) ATPase activity analysis of the DppBCDF variants under different conditions. The DppBCDF was reconstituted in nanodiscs. Error bars represent mean ± SD based on three independent measurements. The data underlying this figure can be found in S1 Data.

ABC transporters use a canonical “alternating-access” mechanism for substrate transport across the membrane [14,15,36]. To investigate the substrate translocation mechanism, we attempted to obtain DppBCDF in other conformational states. To achieve this, we mutated Glu179 (E179) of DppD and Glu187 (E187) of DppF, two key catalytic residues for ATP hydrolysis, into glutamine residues (DppD^E179Q^ and DppF^E187Q^). These mutations prevent ATP hydrolysis without affecting ATP binding. By incubating the inert ATP analogs, AMPPNP or ATPγS, and Mg^2+^ with the complex for 30 min in ice prior to cryo-EM sample preparation, we determined the ATPγS (or AMPPNP)-bound structures of the mutant DppBCD^E179Q^F^E187Q^. (S7A–S7H Fig), which we refer to as ATPγS (or AMPPNP)-DppBCDF unless otherwise specified. Structures reveal that both ATPγS-DppBCDF and AMPPNP-DppBCDF adopt an inward-facing conformation, nearly identical to that of apo-DppBCDF (Figs 2A and S8A). The RMSD between the ATPγS-DppBCDF and apo-DppBCDF is only 0.27 Å for 1,112 aligned Cα atoms. Intriguingly, although sequence alignment and structure superposition indicate that DppD, similar to DppF, does possess functional ATP binding-and-hydrolysis motifs [37–40] (Figs 2B and S8B), ATP analogues are only bound with DppF but not DppD in both ATPγS-DppBCDF and AMPPNP-DppBCDF (Fig 2B). This unusual structural feature motivated us to check the ATPase activity of DppBCDF. Consistent with our structural findings, DppBCDF has essentially no ATPase activity, even in the presence of dipeptide substrates such as Ala-Ala (AA) (Fig 2C). In sharp contrast, high ATPase activity is observed when DppA was supplied in the assay, and dipeptide AA can substantially potentiate its ATPase activity (Fig 2C). Intriguingly, while DppABCD^E179Q^F^E187Q^ has no ATPase activity as expected, both DppABCD^E179Q^F and DppABCDF^E187Q^ have evident and similar ATPase activity (Fig 2C), indicating that in the presence of DppA, both DppD and DppF can bind-and-hydrolyze ATP. Taken together, the ATPase activity assays show that DppBCDF alone lacks ATPase activity, presumably because a single ATP binding to DppBCDF is insufficient to induce its conformational switch. it is probable that DppA binding causes subtle conformational changes of DppBCDF, enabling both DppD and DppF to bind and hydrolyze ATP, thereby activating the DppBCDF translocator.

### Overall structure of the ATPγS-bound DppABCDF complex

**Fig 3:**
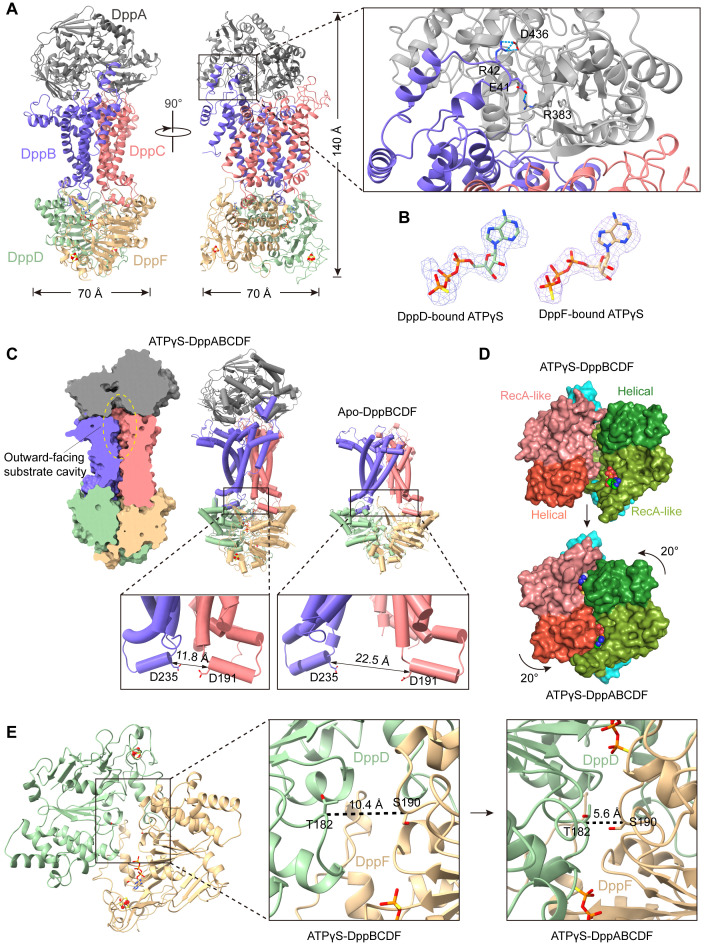
The overall structure of ATPγS-DppABCDF. (**A**) Cartoon representation of the ATPγS-DppABCDF complex structure from two viewpoints. Color scheme for DppB, DppC, DppD and DppF is same as in Fig 1 and DppA is colored gray. Residues E41 and R42 in the scoop loop of DppB form salt bridges with residues R383 and D436 of DppA, respectively. (**B**) EM densities of two ATPγS molecules bound with DppD (left) and DppF (right) in the DppABCDF structure. (**C**) Structural comparison of ATPγS-DppABCDF and apo-DppBCDF. Clipped view of the outward-facing substrate loading cavity in ATPγS-DppABCDF (left), the outward-facing conformation of ATPγS-DppABCDF (middle) and inward-facing conformation of apo-DppBCDF (right). Two inserts showing distance changes of the two coupling helices in two states. (**D**) Surface representations of the ATPases in the two states viewed from membrane plane. The RecA-like domain and the helical subdomain are differentiated by color shadings. The rotation of the helical subdomain with respect to the RecA-like domain during transition from the inward-facing conformation (ATPγS-DppBCDF) to the outward-facing conformation (ATPγS-DppABCDF) is indicated. (**E**) Comparison of DppBCDF ATPase (middle) and ATPγS-DppABCDF ATPase (right). The Cα-Cα distances between T182 in DppD and S190 in DppF are indicated.

Next, we wanted to know how and why DppA is able to activate the DppBCDF translocator. To explore this, we attempt to obtain the cryo-EM structure of DppABCDF. Unlike *Mtb*DppABCD and *Mtb*OppABCD in which their SBPs, *Mtb*DppA and *Mtb*OppA, stably bind to their respective translocators during the transport cycle. We were unable to co-purify the complete DppABCDF complex using tagged DppA and DppBCDF. When DppA and DppBCDF, purified separately, were mixed and analyzed by size-exclusion chromatography, DppA and DppBCDF eluted at different peaks. As a result, we were unable to obtain the wild-type DppABCDF pentameric complex. However, when purifying DppBCD^E179Q^F^E187Q^, we noticed that the DppBCD^E179Q^F^E187Q^ complex pulled down a slight amount of endogenous DppA in *E. coli* (S9A Fig). This prompted us to express DppA_His_BCD^E179Q^F^E187Q^ (DppA C-terminus His-tag) to obtain sufficient amount of protein complex with correct stoichiometry for structural study (S9B and S9C Fig). Using this strategy, we determined the ATPγS-bound DppABCD^E179Q^F^E187Q^ complex structure at 2.73 Å by addition of ATPγS and Mg^2+^ prior to cryo-EM sample preparation (S9D–S9G Fig), and we refer to this structure as ATPγS-DppABCDF unless otherwise specified. Structure reveals that DppABCDF forms a compact pentameric structure with an approximate dimension of 70 × 70 × 140 Å (Fig 3A). A DppA molecule caps at the top of the two integral membrane proteins, DppB and DppC, in the periplasm, and two ATPases, DppD and DppF, adopt a closed conformation in the cytosol (Fig 3A).

### DppBCDF adopts an outward-facing conformation in ATPγS-DppABCDF

Unlike apo-DppBCDF and ATPγS (or AMPPNP)-DppBCDF, which adopt an inward-facing conformation, ATPγS-DppABCDF adopts an outward-facing conformation with two ATPγS molecules bound at the interface of the two NBDs (Fig 3A and 3B). In ATPγS-DppABCDF, a striking rearrangement of the TM helices of both DppB and DppC, which form the substrate translocation pathway, results in the opening of the periplasmic substrate entry gate (Fig 3C). Specifically, the periplasmic substrate entry gate is open to approximately 9.0 Å in diameter in ATPγS-DppABCDF. DppA, DppB and DppC together form an outward-facing cavity along the substrate translocation pathway, extending to the middle of the membrane (Fig 3C). This implies that in this state, dipeptide substrate should retain in the outward-facing cavity rather than being transported across the membrane. In line with the conformational changes of the substrate translocation pathway, the Cα–Cα distance between two residues D235^DppB^ and D191^DppC^ that locates in two coupling helices shortens to 10.7 Å in ATPγS-DppABCDF from 22.5 Å in DppBCDF (Fig 3C). Additionally, distinct from ATPγS (or AMPPNP)-DppBCDF, in which only one ATPγS (or AMPPNP) molecule binds to DppF, two NBDs, DppD and DppF, in ATPγS-DppABCDF adopt a closed conformation with two ATPγS molecules bound at the interface of between DppD and DppF (Fig 3A–3C). The two ATPγS molecules act as molecular glues, shortening the Cα–Cα distance between the D-loop T182^DppD^ and the opposite D-loop S190^DppF^ in the RecA-like subdomain from 10.4 to 5.6 Å (Fig 3E). Additionally, the transition from the inward-facing to the outward-facing conformation involves a rotation of approximately 20° in the helical subdomain (Fig 3D). Taken together, these findings suggest that concurrent binding of DppA and ATP to DppBCDF does induce conformational changes of translocator, and the ATPγS-DppABCDF structure snapshots a typical pre-catalytic (or ATP-bound) state of the transporter.

Structural overlay of the pre-catalytic DppABCDF with *Mtb*DppABCD and *Mtb*OppABCD reveals that the overall structure of the ABC translocators is similar, yet the scoop motif and the short α3 helix of the ABC translocators and SBPs exhibit significant deviations (S10 Fig).

### Functional roles of the periplasmic scoop motif of DppB in dipeptide substrate import

**Fig 4:**
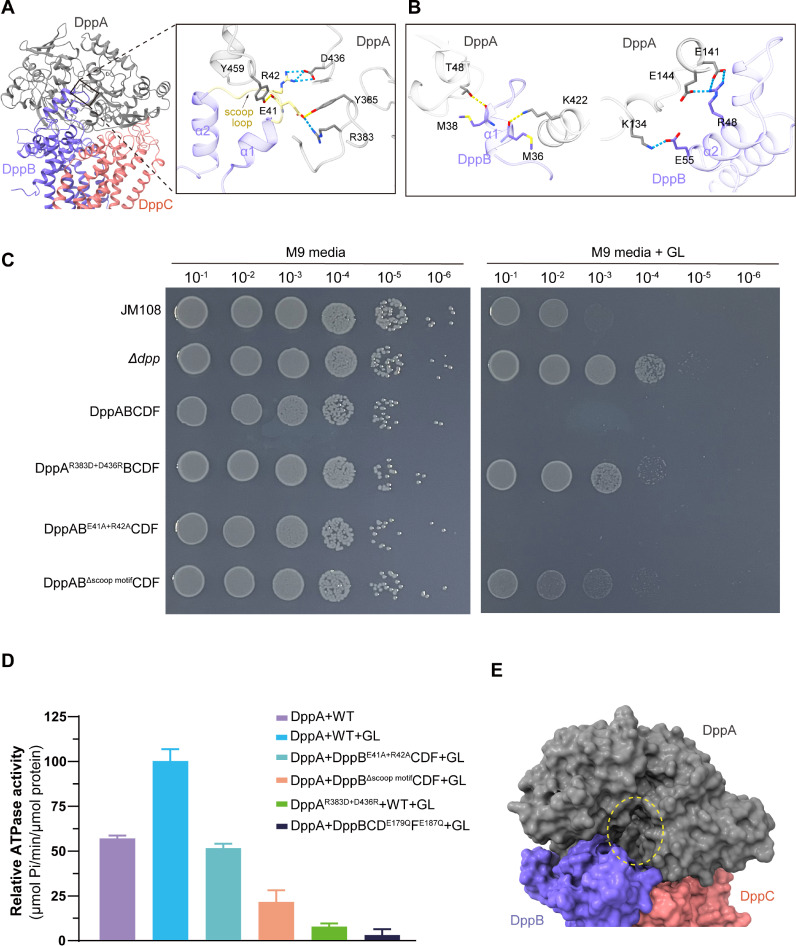
Close-up view of the interactions between DppA and the scoop motif of DppB, along with the functional characterization of the scoop motif. (**A**) Close-up view of the interactions between DppA and the scoop loop in the scoop motif of DppB. (**B**) Close-up view of interactions between DppA and α1 (left) or α2 (right) of the scoop motif. (**C**) Growth phenotypes of the Δ*dpp E. coli* JM108 strain harboring plasmid over-expressing DppABCDF variants on M9 medium agar plate that was supplemented with (left) or without (right) 0.15 mM dipeptides GL. All of the complementation assays were repeated at least three times and a representative result is shown. (**D**) ATPase activity analysis of DppBCDF variants under different conditions. Error bars represent mean ± SD based on three independent measurements. (**E**) Surface representation showing the scoop motif in DppB acts as a door sealing the outward-facing substrate cavity. Deletion of the scoop motif in DppB could result in the released dipeptides escaping into the periplasm rather being transported across the membrane. The data underlying this figure can be found in S1 Raw Images and S1 Data.

Type I ABC importers commonly use a periplasmic “scoop loop” protruding from one of the TMDs to interact with their respective SBPs for substrate release [16,17,39,41]. In DppBCDF and ATPγS (or AMPPNP)-DppBCDF, the periplasmic scoop motif region in DppB is invisible due to its conformational flexibility without interacting partner. However, in ATPγS-DppABCDF, the DppB scoop motif, which consists of two short α helices (α1 and α2) connected by a scoop loop, is clearly resolved (Fig 4A). The DppB scoop motif binds and occupies the cleft between the N-lobe and C-lobe of DppA (Fig 4A and 4B). In particular, its α1 helix and α2 helix interact with the C-lobe and the N-lobe of DppA, respectively, whereas the side chains of two conserved charged residues, E41^DppB^ and R42^DppB^, from the scoop loop form two salt bridges with side chains of residues R383^DppA^ and D436^DppA^ (Fig 4A). The side chains of residues R383^DppA^ and D436^DppA^ play critical roles in dipeptide capture by forming charge–charge interactions with the carboxyl and amino groups of the bound dipeptide (S1B Fig). The scoop motif, therefore, may prevent the dipeptide substrate from binding back-and-forth to DppA after its release. On the other hand, in ATPγS-DppABCDF, DppA adopts a semi-open conformation, distinct from the crystal structures of apo-DppA and Gly-Leu (GL)-bound DppA (S11 Fig). Taken together, concurrent binding of DppA and ATP to DppBCDF causes release of the dipeptide substrate from DppA into the outward-facing cavity that is surrounded by DppA, DppB and DppC, and this process appears to do not require ATP hydrolysis. Because the binding mode of the DppB scoop motif seals the outward-facing cavity, we propose that the DppB scoop motif may play important roles in ensuring peptide import by preventing the released dipeptide from back-and-forth re-binding to DppA and averting dipeptide escaping into periplasm upon being released from DppA via forming a sealed outward-facing substrate cavity.

To probe functional importance of the DppB scoop motif and attest our hypothesis, we performed bacterial complementation assays and measured the ATPase activities for various DppABCDF variants. Previous studies imply that accumulation of peptides containing leucine residues is toxic to *E. coli*, and strains with impaired peptide transporters become resistant to this toxicity [42]. To conduct complementation assays, we first constructed the *dppABCDF*-deleted (Δ*dpp*) *E. coli* JM108 strain and test if the Δ*dpp* JM108 strain is tolerant to high concentrations of dipeptide GL. As expected, the growth of wild-type *E. coli* JM108 was evidently impaired on M9 medium agar plate supplemented with 0.15 mM dipeptides GL, whereas the Δ*dpp* JM108 strain grew normally under the same condition (Fig 4C). This demonstrates that deletion of *dppABCDF* genes does not affect the growth of *E. coli* JM108 strain, yet intracellular accumulation of GL via endogenous DppABCDF are harmful to wild-type *E. coli* JM108. In line with this rationale, the Δ*dpp E. coli* JM108 strain harboring a plasmid that overexpresses DppABCDF caused cell death (Fig 4C), suggesting that the DppABCDF transporter efficiently uptakes GL in Δ*dpp E. coli* JM108 strain once overexpressed. Therefore, we can use this complementation experiment to assess the GL uptake capability of various DppABCDF variants. As shown in Fig 4C, the Δ*dpp* JM108 strain over-expressing the charge-reversal mutations at the substrate binding site of DppA, DppA^R383D+D436R^BCDF, grew normally, indicating that the DppA^R383D+D436R^BCDF transporter, which lost capability to capture dipeptides, failed to transport GL into Δ*dpp* JM108 strain (Fig 4C). Indeed, using isothermal titration calorimetry (ITC), no binding affinity was detected between DppA^R383D+D436R^ and dipeptides GL or AA (S12A and S12B Fig). Consistent with this phenotype, even in the presence of substrate GL, the ATPase activity of DppA^R383D+D436R^BCDF is only one-tenth of that of the wild-type DppABCDF (Fig 4D). The substantial decrease of ATPase activity of DppA^R383D+D436R^BCDF might result from at least two potential accumulative causes: First, DppA^R383D+D436R^does not bind dipeptide substrates, so DppA^R383D+D436R^BCDF lacks substrate stimulation effects on its ATPase activity. Second, it is likely that apo-DppA^R383D+D436R^, which adopts an open conformation, may have a lower affinity to DppBCDF as compared to that of the clamped dipeptide-bound DppA, especially for DppA^R383D+D436R^ that additionally loses two charge–charge interactions to the scoop loop of the DppB scoop motif. In sharp contrast, Δ*dpp* JM108 strain that expresses the DppAB^E41A+R42A^CDF mutant transporter did not grow under the same condition (Fig 4C), indicating that the scoop loop in DppB is not critical for substrate release from DppA, but it may play a more important role in preventing back-and-forth re-binding to DppA upon released. In line with this, in the presence of GL, the ATPase activity of DppAB^E41A+R42A^CDF is approximately half that of the wild-type DppABCDF, significantly higher than that of DppA^R383D+D436R^BCDF (Fig 4D). However, the growth of Δ*dpp* JM108 strain that harbors the DppAB^Δscoop-motif^CDF mutant transporter was apparently impaired, and the ATPase activity of DppAB^Δscoop-motif^CDF is about one-fourth of that of the wild-type DppABCDF (Fig 4D). These observations imply that DppAB^Δscoop-motif^CDF does not efficiently transport GL like DppAB^E41A+R42A^CDF. A plausible cause is that DppAB^Δscoop-motif^CDF is unable to form a sealed outward-facing substrate cavity, allowing the released substrates can readily escape into periplasm before being transported across the membrane. Taken together, the striking differences in phenotype and ATPase activity between DppA^R383D+D436R^BCDF and DppAB^E41A+R42A^CDF also manifest that the DppBCDF translocator prefers to binding substrate-bound DppA, thus avoiding futile transport cycle caused by apo-DppA binding under physiological conditions. These observations indicate that a functional DppA is essential for dipeptide uptake and that the DppB scoop motif may play a role in preventing the released dipeptide substrate from escaping into the periplasm (Fig 4E).

## Discussion

**Fig 5:**
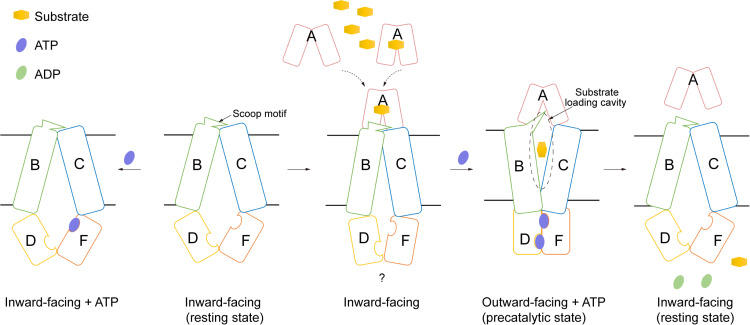
A proposed working model for the Dpp dipeptide importer. Diagram of the “alternating-access” mechanism for the DppABCDF dipeptide transporter. In this model, DppA (or dipeptide-bound DppA)-bound DppBCDF structure is unavailable.

In this work, we present cryo-EM structures of both DppBCDF, the translocator, and DppABCDF, the full transporter, in different states. Combined with enzymatic assays and complementation experiments, we conclude that DppA, the peptide-binding protein, is crucial for activating the DppBCDF translocator. Different from the recently reported peptide importers *Mtb*DppABCD and *Mtb*OppABCD, where DppA/OppA, the SBPs, stably bind to the translocator during the peptide transport cycle, both apo-DppA and dipeptide-bound DppA possibly have only low affinities to apo-DppBCDF translocator. DppA forms a stable complex with DppBCDF only in the ATP-bound state, *i.e.*, the precatalytic state. This indicates that the periplasm-localized DppA not only can carry dipeptide substrates from a distant region in the periplasmic space to the translocator, but also can stay bound transiently with the translocator waiting for dipeptide substrates to feed for transport. By contrast, in *M. tuberculosis* and Gram-positive bacteria, SBPs have to anchor to the membrane to prevent diffusion into the external environment. Their tight interactions with the translocator also enhance the efficiency of the peptide uptake, as membrane-anchored SBPs could be inefficient in accessing the translocator via lateral diffusion in the membrane. Furthermore, the dipeptide translocator DppBCDF alone does not hydrolyze ATP, which avoids futile consumption of cellular ATP. On the basis of our studies, we propose a working model for dipeptide transporters in Gram-negative bacteria (Fig 5). In the resting state, the substrate translocation pathway of DppBCDF is inward-facing open to the cytoplasm, sealing the membrane. In this state, binding of a single ATP molecule to DppF in DppBCDF does not induce conformational changes, and DppBCDF does not consume intracellular ATP. The binding of apo-DppA or dipeptide-bound DppA to DppBCDF likely induces subtle conformational changes in the entire translocator, enabling the two ATPases, DppD and DppF, to simultaneously bind and hydrolyze ATP. The concurrent binding of DppA and ATP to DppBCDF leads to significant conformational changes, causing DppABCDF to adopt an outward-facing conformation. In this pre-catalytic state, if DppA carries a dipeptide substrate prior to binding to DppBCDF, the substrate should be released into the sealed outward-facing substrate cavity. Following the pre-catalytic state, the hydrolysis of two ATP molecules into ADP by DppD and DppF provides the energy to induce the DppBCDF translocator into an inward-facing conformation, thereby completing the dipeptide transport across the membrane. In this state, apo-DppA has a lower affinity for the inward-facing open DppBCDF and could diffuse away from the translocator.

While the structure of ATPγS-DppABCDF snapshots a pre-catalytic state of the DppABCDF transporter, we did not observe any substrate cryo-EM density in the outward-facing substrate cavity. Classical type I ABC transporters represented by maltose transporter reveal specific substrate binding sites within the translocation channel [39,43]. Indeed, in the pre-catalytic state structures of both *Mtb*DppABCD and *Mtb*OppABCD, no substrate cryo-EM densities were observed as well [16,17]. Several reasons may account for this difference. First, neither the Dpp nor Opp peptide transporters are highly specific substrate transporters, so they may lack specific binding sites within the translocation pathway. Second, both DppABCDF and *Mtb*DppABCD/*Mtb*OppABCD were co-purified complexes, so they may bind a mixture of peptide substrates [27–29,44], which could lead to averaging of substrate density during cryo-EM classification. Third, since apo-DppA may bind to DppBCDF, the substrate density within the outward-facing cavity could be further averaged during calculation. In summary, the structures of DppABCDF from *E. coli* provide a framework for further investigation into the transport mechanisms of ABC peptide transporters and offer a structural basis for development of antibiotics targeting Gram-negative bacterial pathogens.

## Materials and methods

### Expression and purification of Dpp proteins

The DppABCDF operon, including a DNA fragment encoding a C-terminal fusion Strep-tag II, was ligated into the pBAD22 vector. The construct was transformed into BL21 competent cells and induced with 0.05% (w/v) l-arabinose (Lablead, Beijing) at 26 °C for 12 h for over-expression. The cells were harvested and resuspended in 20 mM Tris-HCl pH 7.5, 150 mM NaCl buffer, followed by cell lysis using high-pressure homogenization (ATS Engineering, Shanghai). Crude membranes were collected by centrifugation and homogenized in a buffer containing 20 mM Tris-HCl pH 7.5, 150 mM NaCl, 5% (v/v) glycerol (Sigma), 1% (w/v) n-Dodecyl-β-d-maltopyranoside (DDM, D310S, Anatrace) and 1 mM DTT (Sangon Biotech). The supernatant was incubated with Streptactin Beads 4FF (Smart-Lifesciences) at 4 °C for 1 h, followed by washing with a buffer containing 20 mM Tris-HCl pH 7.5, 150 mM NaCl, 0.1% DDM and 1 mM DTT. The target proteins were eluted using a buffer containing 20 mM Tris-HCl pH 7.5, 150 mM NaCl, 0.1% DDM, 2 mM DTT, and 2.5 mM d-biotin disulfide (Sigma). Further purification of the target proteins was achieved using a Superdex 200 gel filtration column (10/300, GE Healthcare) in a buffer containing 20 mM Tris-HCl pH 7.5, 150 mM NaCl, 0.006% GDN (GDN101, Anatrace) and 2 mM DTT.

To avoid contaminations from endogenous proteins during the purification of mutated proteins, protein expression was performed using a *Δdpp E. coli* strain. For the expression and purification of the pentameric complex used for cryo-EM structural analysis, the DppA_His_BCD^E179Q^F^E187Q^_strep_ construct was utilized, with a His-tag fused to the C-terminus of DppA. Affinity purification was carried out using Ni-NTA resin (GE Healthcare), with the buffer additionally containing imidazole (Sigma). Purification of the soluble fraction of DppA involved collecting the supernatant after centrifugation of the disrupted cells. The Ni-NTA resin was then washed using a stepwise decrease in concentration of 2 M guanidine hydrochloride over 10 column volumes to effectively remove endogenous peptide substrates [45,46].

### Construction of the Δ*dpp E. coli* strain

The λ-red system was used to knock out the *dpp* operon from the genome of *E. coli* K12 derivative strain JM108 (*endA1 recA1 gyrA96 thi-1 relA1 glnV44 Δ(lac-proAB) hsdR17* (rK- mK+)) [47]. Specifically, 1,000 bp gene fragments upstream and downstream of the *dpp* operon were cloned as homologous arms and ligated to the 5′ and 3′ ends of the chloramphenicol resistance gene. The construct was then transformed into JM108 electrocompetent cells containing pKD46 and plated on chloramphenicol-resistant plates. After 20 h of growth at 30 °C, colonies were observed, and single clones were picked and grown at 30 °C with shaking at 200 rpm. Polymerase Chain Reaction (PCR) was performed using primers designed to span the homologous arm sequences, and the amplified products were sequenced after gel electrophoresis to confirm the deletion of the *dpp* operon sequence and replacement with the chloramphenicol resistance gene, indicating successful knockout.

### DppBCDF nanodisc reconstitution

The DppBCDF complex was mixed with MSP1E3D1 and POPG (Avanti Polar Lipids) at a molar ratio of 1:2.4:80 [48]. The mixture was incubated at 4 °C with gentle rotation for 1 h. Bio-beads SM2 (Bio-Rad) were added every 30 min, four times in total, to remove detergents. The sample was then incubated overnight at 4 °C to complete the detergent removal process. After removing the Bio-beads by low-speed centrifugation, the sample was loaded onto a Superdex 200 gel filtration column using a buffer containing 20 mM Tris-HCl pH 7.5, 150 mM NaCl, and 2 mM TCEP (Beyotime). Fractions corresponding to the peak of the complex were collected and concentrated for enzyme activity assays.

### ATPase activity assay

ATPase hydrolysis activity was detected using Malachite Green Phosphate Assay Kits (Sigma). The reaction mixture contained 0.02 μM of DppBCDF, 0.1 μM of DppA, 0.2 μM of substrate AA or GL, 0.2 μM of ATP and 0.5 μM of MgCl_2_. The system was supplemented with 80 μL of 20 mM Tris-HCl pH 7.5, 150 mM NaCl and 2 mM TCEP buffer. The reaction proceeded at 37 °C for 15 min, followed by the addition of 20 μL of stop solution and incubation at room temperature for 30 min for color development. The samples were transferred to a 96-well plate in advance, and the absorbance at 620 nm was measured using a spectrophotometer (EnSpire). The amount of phosphate produced in the reaction was calculated from a standard curve and converted into ATP hydrolysis activity. The experiment was performed in triplicate, and the results were analyzed using GraphPad Prism 10.

### ITC assay

0.2 mM of substrate peptide and 0.02 mM of protein were prepared in the same buffer (20 mM Tris-HCl pH 7.5, 150 mM NaCl). The titration experiment was performed on an Affinity ITC LV (Waters TA) instrument with a stirring speed of 125 rpm and a temperature of 25 °C. The data obtained from the titration were analyzed using the ITC analysis software.

### Preparation and data collection of frozen samples

DppBCDF sample at a concentration of 5 mg/mL were applied onto Quantifoil Cu 300 mesh R1.2/1.3 grids for glow-discharged (H_2_/O_2_, 60 s) (Solarus 950, Gatan), blotting 4 μL of the protein solution. The grids were then incubated for 5 s at 16 °C and 75% humidity in a Leica EMGP plunge-freezing apparatus before blotting for 3.5 s and plunge-freezing into liquid ethane to prepare frozen samples. For sample DppBCD^E179Q^F^E187Q^ at 5 mg/mL, 2 mM ATPγS or AMPPNP, and 2 mM MgCl_2_ were added and incubated on ice for 30 min before immediate preparation of frozen samples using the same conditions. Similarly, sample of DppABCD^E179Q^F^E187Q^ with 2 mM ATPγS and 2 mM MgCl_2_ were incubated on ice for 30 min before being frozen. Due to significant air-liquid interface issues with our samples, we used graphene oxide-coated grids, specifically Quantifoil Cu 300 mesh R2/1 grids covered with graphene oxide (Sigma) [49]. These grids were incubated for 60 s under the same conditions. Single-particle cryo-EM data were collected on a 300 kV Titan Krios G2/G3i (Thermo Fisher Scientific) equipped with a K2 Summit (Gatan) and a GIF Quantum Model 967 energy filter. Data collection was automated using the beam-image shift method in the SerialEM program [50,51], with super-resolution mode and a pixel size ranging from 0.41 to 0.52 Å, while the defocus was set between −1.2 and −2.5 μm, and a total electron dose of 40–60 e^−^/Å² was distributed across 32 frames recorded in movie mode.

### Cryo-EM data processing

The process of cryo-EM data processing is summarized in the Extended Data and was performed using cryoSPARC [52]. The movie stacks underwent Patch-based motion correction and were dose-weighted with 2-fold binning. Micrograph contrast transfer functions were estimated using Patch-based CTF estimation. Particle picking was conducted using blob picker, and particles were extracted with a box size ranging from 256 to 384 pixels. Subsequently, several rounds of 2D classification were performed, and particles from selected 2D classes were subjected to Ab-initio reconstruction, with all classes used for heterogeneous refinement. Particles from the best classes were employed for non-uniform refinement [53], and local refinement was utilized to enhance map quality. For all final maps, local resolution was computed, and sharpening was conducted using deepEMhancer [54].

### Model building and refinement

The building of the structural model was completed using ModelAngelo [55], and density maps were fitted with structural models in ChimeraX [56]. Subsequently, sharpened maps were used for adjustments in WinCoot 0.9.8.1 and ISOLDE [57,58], while original maps were utilized for real-space refinement in Phenix [59]. The models were evaluated in MolProbity [60]. A summary of the statistics for data collection and structure refinement is provided in S1 Table. High-resolution images for publication were prepared by using PyMOL (Schrödinger, LLC) and ChimeraX.

## Supporting information

S1 Fig Substrate-capture via a proposed “Venus flytrap” mechanism by SBPs. (A) Cartoon representation of apo-DppA (PDB code: 1dpe) and dipeptide (Gly-Leu, GL)-bound DppA (PDB code: 1dpp). GL shown in a sphere mode. DppA consists of an N-lobe (salmon), hinge region (blue) and a C-lobe (light green). (**B**) Close-up view of the GL-DppA interactions, the GL forms two salt bridges with R383 and D436 of DppA, as shown in the right panel.
(TIF)

S2 Fig Expression, purification, and cryo-EM data processing workflow of the DppBCDF complex. (A) Schematic diagram of the *dpp* operon in *E. coli* genome. (**B**) Size-exclusion chromatography and SDS-PAGE analysis of the purified DppBCDF complex. The peak fraction (P1) in size-exclusion chromatography was analyzed by SDS-PAGE (12%). (C) Representative raw micrograph of the DppBCDF complex and schematic representation of the processing workflow. (**D**) Final deepEMhancer-postprocessed map colored according to the local resolution estimation in cryoSPARC. (**E**) Fourier shell correlation (FSC) curves of DppBCDF. (**F**) Angular distributions of DppBCDF. The data underlying this figure can be found in S1 Raw Images.
(TIF)

S3 Fig EM densities of TMs in DppB and DppC in the DppBCDF structure. (**A**) EM densities of TM1–6 of DppB (slate blue). (**B**) EM densities of TM1–6 of DppC (light coral). The conformation of TM1 in DppB and DppC is strikingly different.
(TIF)

S4 Fig Residues involved in DppB-DppD interactions and DppC-DppF interactions are highly conserved in different bacterial strains. (**A**) Sequence alignment of DppB and DppD in different bacterial strains (*E. coli*, *P. aeruginosa*, *H. influenzae*, *Y. pestis*, *K. pneumoniae*, *S. enterica*, *S. flexneri*). Residues D235, T239, K243, H254 and N258 of DppB form polar interactions with residues T100, S101, N103 and E114 of DppD, with blue representing amino acids that form salt bridges and yellow representing hydrogen bonds. These residues are identical in different bacterial strains. (**B**) Sequence alignment of DppC and DppF in different bacterial strains (*E. coli*, *P. aeruginosa*, *H. influenzae*, *Y. pestis*, *K. pneumoniae*, *S. enterica*, *S. flexneri*). Residues S196, N210 and N214 of DppC form polar interactions with residues N113 and E124 of DppF. These residues are identical in different bacterial strains.
(TIF)

S5 Fig The iron-sulfur cluster-bound domains in DppD and DppF play critical roles in stabilizing the DppBCDF complex. (**A**) 12% SDS-PAGE analysis of the purified DppBCDF variants. Lane 1, affinity-purified wild-type DppBCDF; lane 2, affinity-purified DppBCD^C284S+C290S+C297S+C315S^F; lane 3, affinity-purified DppBCDF^C293S+C299S+C306S+C323S^. Cys-to-Ser mutation in either DppD or DppF resulted in no pull-down of the DppBCDF complex. (**B**) The purified DppBCDF complex exhibited highly morphological difference in EM micrographs in the absence of DTT. Reducing condition is critical for Cys residues in both DppD and DppF chelating the [4Fe-4S] cluster and stabilizing the overall structure of DppBCDF. The data underlying this figure can be found in S1 Raw Images.
(TIF)

S6 Fig Comparison of DppBCDF and *Mtb*DppABCD/*Mtb*OppABCD structures. Cartoon representation of DppBCDF and *Mtb*DppABCD/*Mtb*OppABCD structures from two viewpoints.
(TIF)

S7 Fig Cryo-EM data processing workflow of the ATPγS (or AMPPNP)-DppBCDF complex. (**A**) Representative raw micrograph and 2D classification of the ATPγS-DppBCDF complex and schematic representation of the processing workflow. (**B**) Final deepEMhancer-postprocessed map colored according to the local resolution estimation in cryoSPARC. (**C**) Fourier shell correlation (FSC) curves of ATPγS-DppBCDF. (**D**) Angular distributions of ATPγS-DppBCDF. (**E**) Representative raw micrograph of the AMPPNP-DppBCDF complex and 2D classification and schematic representation of the processing workflow. (**F**) Final deepEMhancer-postprocessed map colored according to the local resolution estimation in cryoSPARC. (**G**) FSC curves of AMPPNP-DppBCDF. (**H**) Angular distributions of AMPPNP-DppBCDF.
(TIF)

S8 Fig Structural overlay of DppBCDF, ATPγS-DppBCDF and AMPPNP-DppBCDF. (**A**) Structural overlay of DppBCDF (lime), ATPγS-DppBCDF (slate blue) and AMPPNP-DppBCDF (salmon) showing no conformation differences among three structures. (**B**) Sequence alignment of DppD and DppF with ATPase sequences containing consensus sites (TM287, ModC, MetN, MalK) and degenerate site (TM288).
(TIF)

S9 Fig Structure determination process of the ATPγS-DppABCDF complex. (**A**) The affinity-purified DppBCD^E179Q^F^E187Q^ complex pull-downed slight amount of endogenous DppA. (**B**) Co-expression and His-tag affinity-purification of DppABCD^E179Q^F^E187Q^ via DppA-His. (**C**) Gel filtration chromatographic profile of the affinity-purified DppABCD^E179Q^F^E187Q^. (**D**–**G**) Schematic representation of the processing workflow of ATPγS-DppABCDF complex. Representative raw micrograph and 2D classes (D), and final deepEMhancer-postprocessed map colored according to the local resolution estimation in cryoSPARC (E). FSC curve (F) and angular distributions (G) used for the final reconstruction of particles generated by cryoSPARC. The data underlying this figure can be found in S1 Raw Images.
(TIF)

S10 Fig Structural comparison between DppABCDF and *Mtb*DppABCD/*Mtb*OppABCD. Cartoon representation of DppABCDF and *Mtb*DppABCD/*Mtb*OppABCD structures from two viewpoints.
(TIF)

S11 Fig DppA adopts a semi-open conformation in ATPγS-DppABCDF. Apo-DppA adopts an open and substrate accessible conformation (left); DppA adopts a semi-open conformation in ATPγS-DppABCDF (middle); DppA adopts a clamped conformation in GL-bound state (right).
(TIF)

S12 Fig The DppA^R383D+D436R^ mutant does not bind dipeptides. (**A**) ITC measurements of the affinity between wild-type DppA (or DppA^R383D+D436R^) and dipeptides AA. The affinity between wild-type DppA and AA is approximately 13.9 nM (left), while DppA^R383D+D436R^ lost binding affinity to AA (right). (**B**) ITC measurements of the affinity between wild-type DppA (or DppA^R383D+D436R^) and dipeptides GL. The affinity between wild-type DppA and GL is approximately 2.2 μM (left), while DppA^R383D+D436R^ lost binding affinity to dipeptide GL (right). The data underlying this figure can be found in S1 Data.
(TIF)

S1 Table Cryo-EM data collection, refinement and validation statistics. (DOCX)

S1 Data Excel file with all individual numerical values corresponding to the data presented in the main and supporting figures. Corresponding figure numbers are indicated in each Excel worksheet.
(XLSX)

S1 Raw Images A PDF file containing all uncropped gel images and bacterial growth images corresponding to the data presented in the main and supporting figures. The corresponding figure numbers are indicated above each image.
(PDF)

## Abbreviations

ABC: ATP-binding cassette
BN-PAGE: Blue Native-polyacrylamide gel electrophoresis
C-lobe: C-terminal lobe
GDN: glyco-diosgenin
ITC: isothermal titration calorimetry
NBDs: nucleotide-binding domains
N-lobe: N-terminal lobe
PCR: Polymerase Chain Reaction
RMSDs: root mean square deviations
SBP: substrate-binding protein
TM: transmembrane
TMDs: transmembrane domains
