# Supplementary figures and images for: Structural characterization of the ABC transporter DppABCDF in Escherichia coli reveals insights into dipeptide acquisition
Source: PLoS Biol. 2025 Mar 7;23(3):e3003026. doi: 10.1371/journal.pbio.3003026 (PMC12136057; doi:10.1371/journal.pbio.3003026)

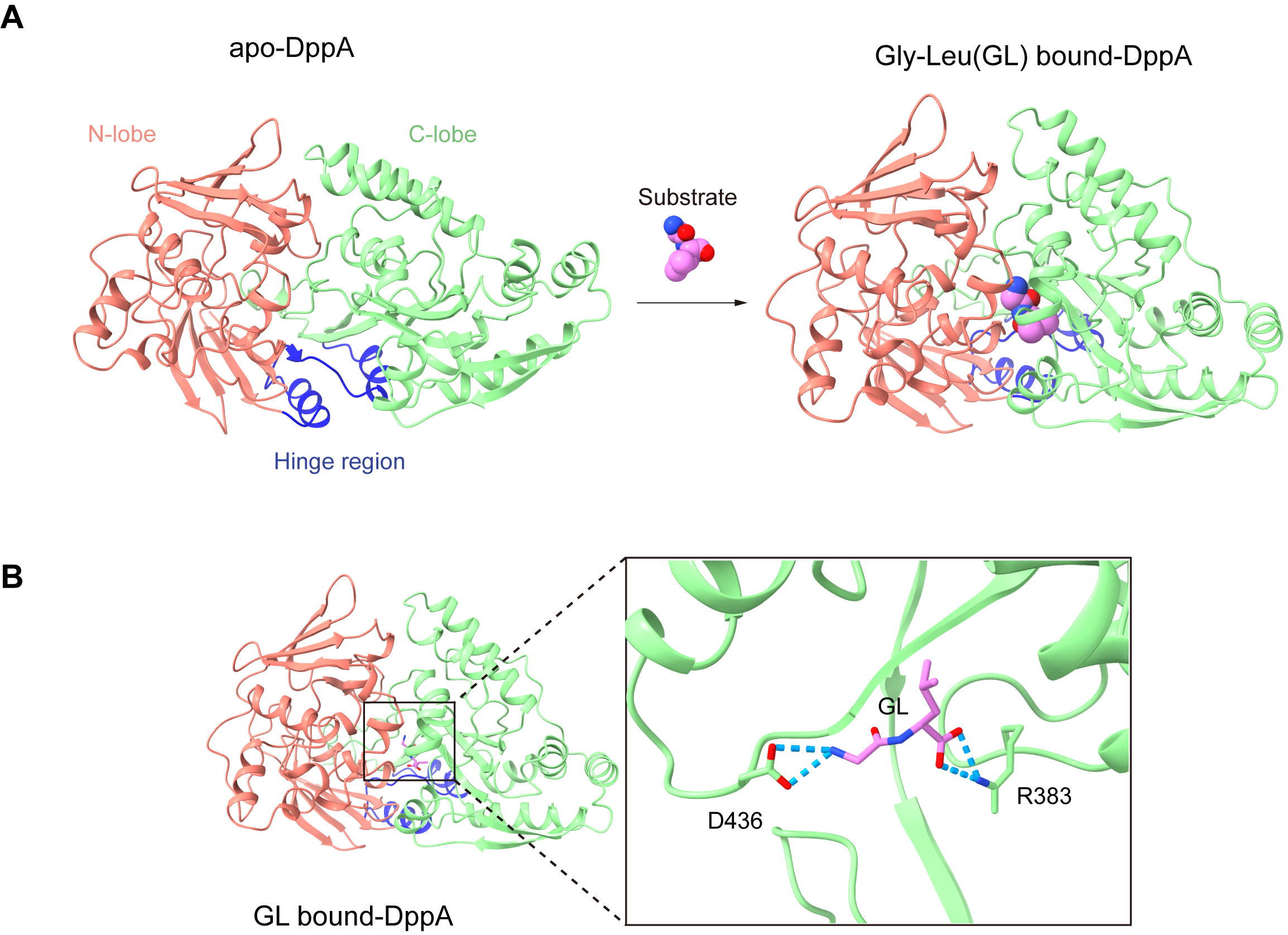

Supplement: S1 Fig — (A) Cartoon representation of apo-DppA (PDB code: 1dpe) and dipeptide (Gly-Leu, GL)-bound DppA (PDB code: 1dpp). GL shown in a sphere mode. DppA consists of an N-lobe (salmon), hinge region (blue) and a C-lobe (light green). (B) Close-up view of the GL-DppA interactions, the GL forms two salt bridges with R383 and D436 of DppA, as shown in the right panel. (TIF) [file pbio.3003026.s001.tif]

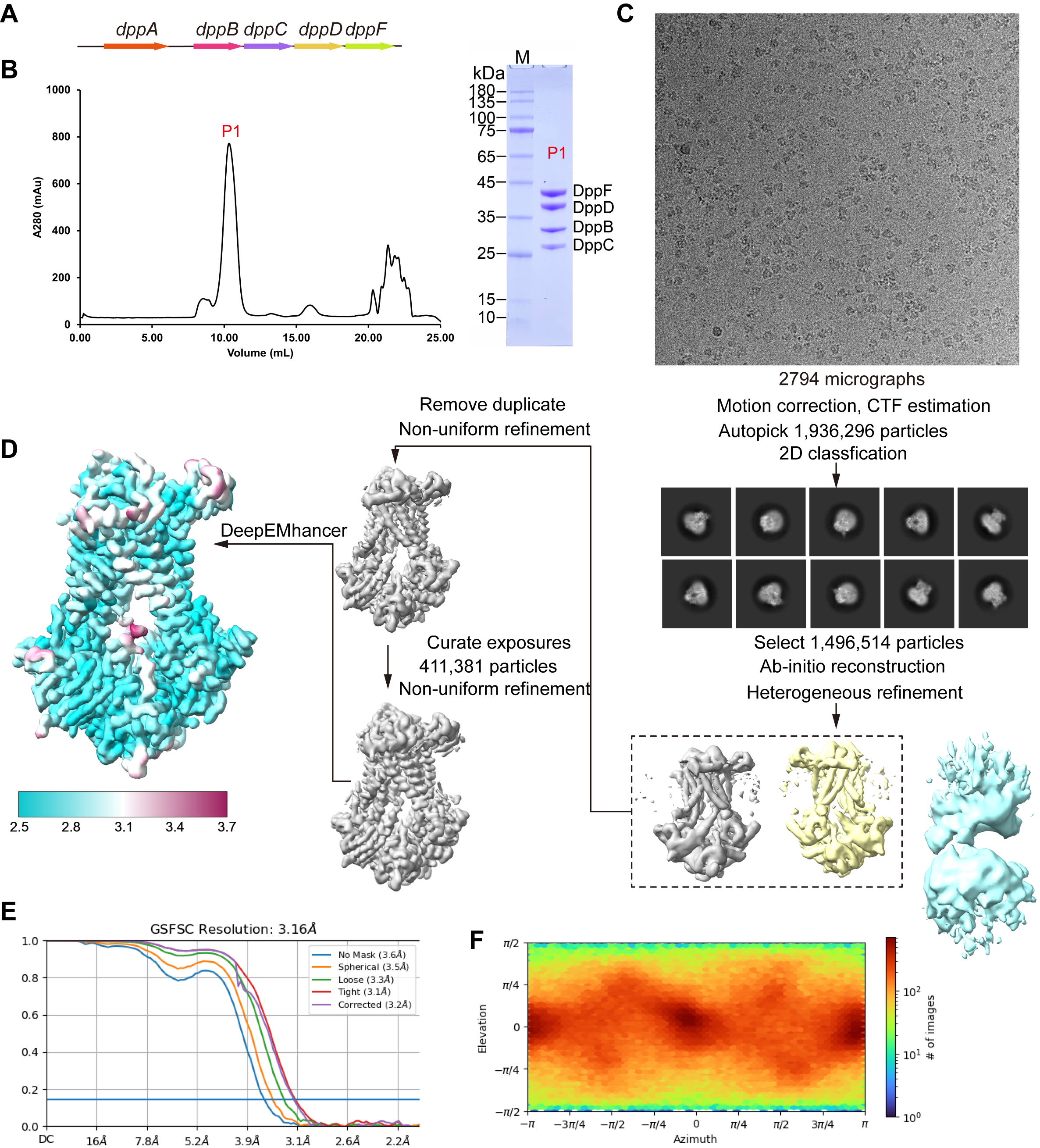

Supplement: S2 Fig — (A) Schematic diagram of the dpp operon in E. coli genome. (B) Size-exclusion chromatography and SDS-PAGE analysis of the purified DppBCDF complex. The peak fraction (P1) in size-exclusion chromatography was analyzed by SDS-PAGE (12%). (C) Representative raw micrograph of the DppBCDF complex and schematic representation of the processing workflow. (D) Final deepEMhancer-postprocessed map colored according to the local resolution estimation in cryoSPARC. (E) Fourier shell correlation (FSC) curves of DppBCDF. (F) Angular distributions of DppBCDF. The data underlying this figure can be found in S1 Raw Images. (TIF) [file pbio.3003026.s002.tif]

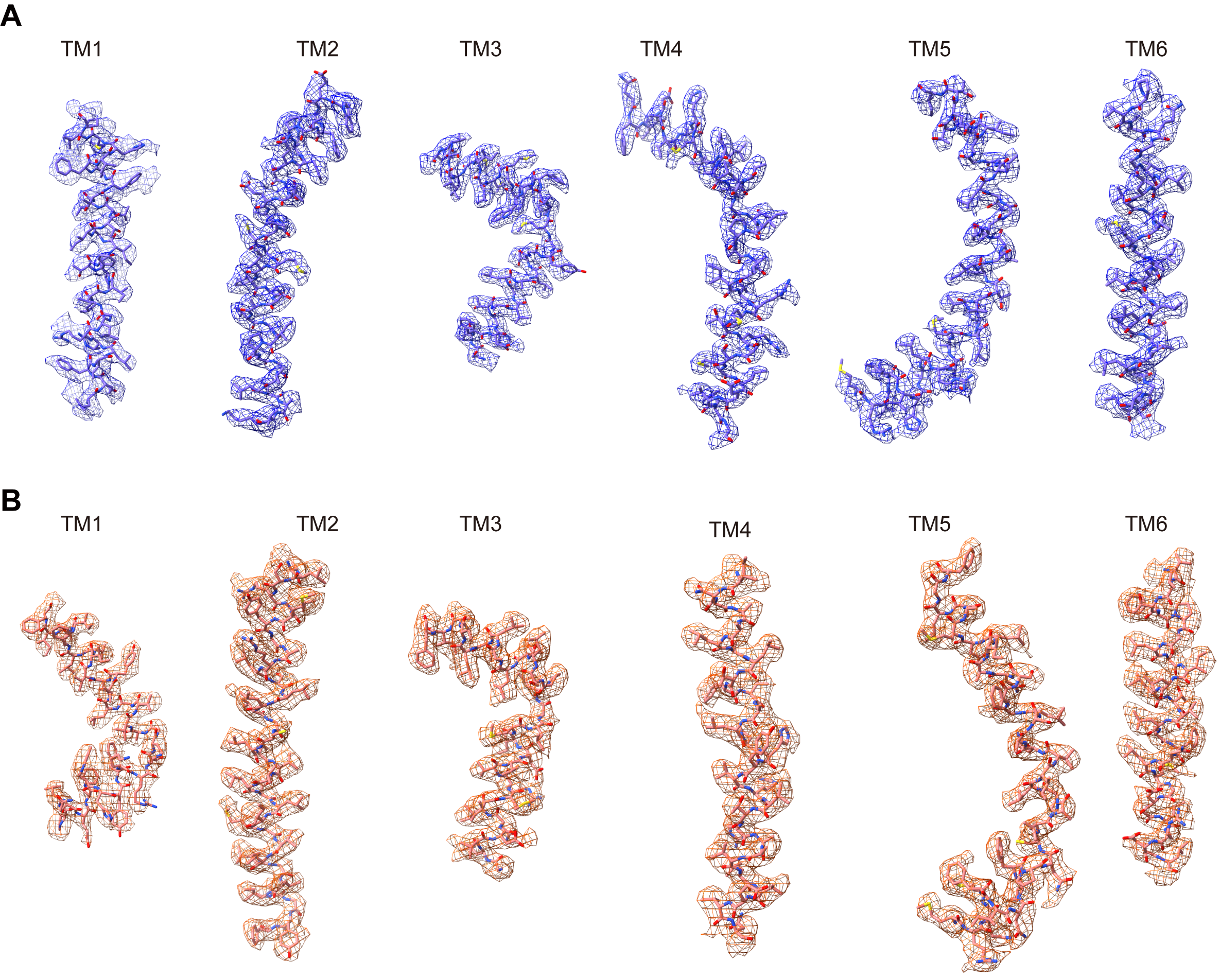

Supplement: S3 Fig — (A) EM densities of TM1–6 of DppB (slate blue). (B) EM densities of TM1–6 of DppC (light coral). The conformation of TM1 in DppB and DppC is strikingly different. (TIF) [file pbio.3003026.s003.tif]

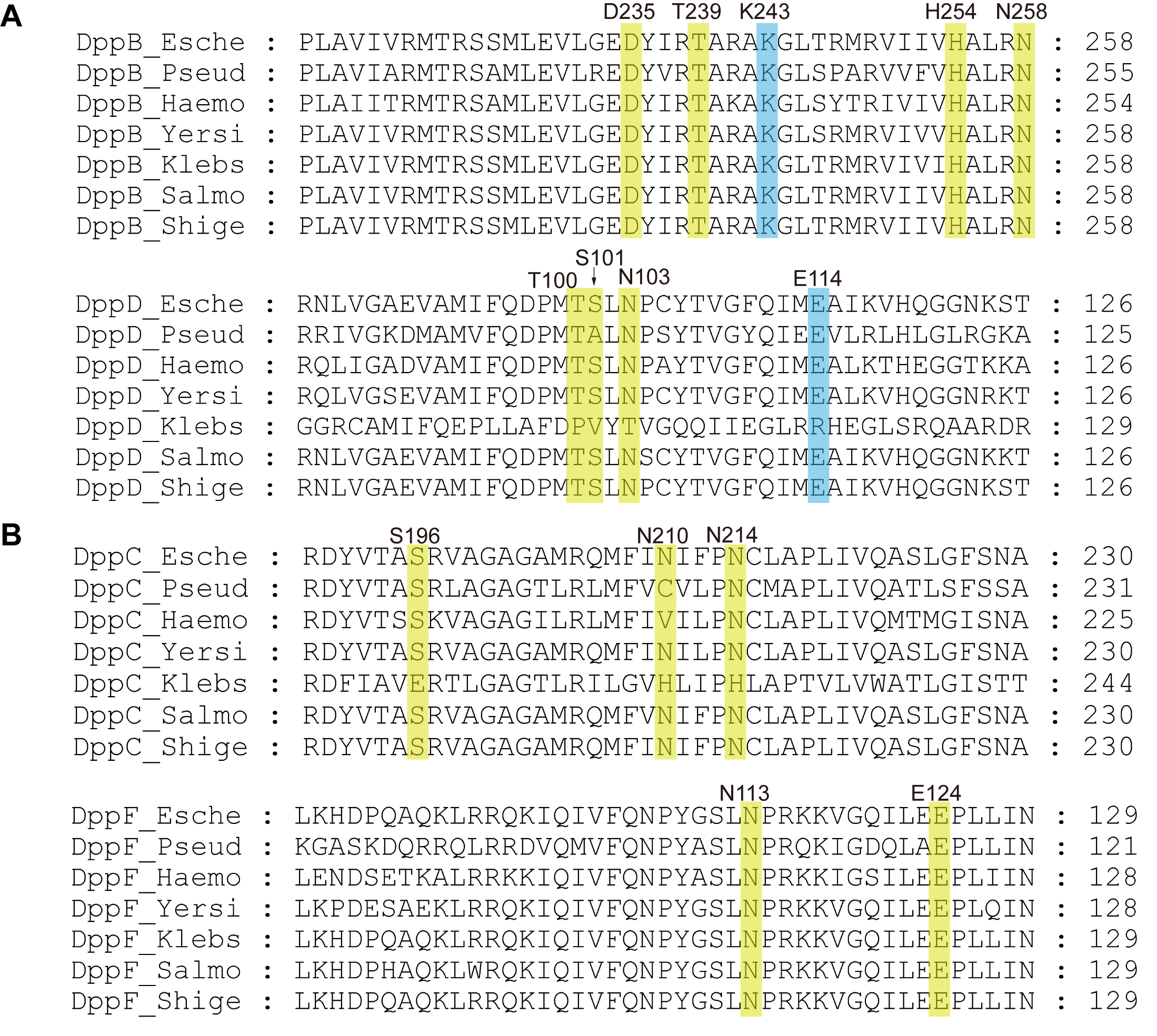

Supplement: S4 Fig — (A) Sequence alignment of DppB and DppD in different bacterial strains (E. coli, P. aeruginosa, H. influenzae, Y. pestis, K. pneumoniae, S. enterica, S. flexneri). Residues D235, T239, K243, H254 and N258 of DppB form polar interactions with residues T100, S101, N103 and E114 of DppD, with blue representing amino acids that form salt bridges and yellow representing hydrogen bonds. These residues are identical in different bacterial strains. (B) Sequence alignment of DppC and DppF in different bacterial strains (E. coli, P. aeruginosa, H. influenzae, Y. pestis, K. pneumoniae, S. enterica, S. flexneri). Residues S196, N210 and N214 of DppC form polar interactions with residues N113 and E124 of DppF. These residues are identical in different bacterial strains. (TIF) [file pbio.3003026.s004.tif]

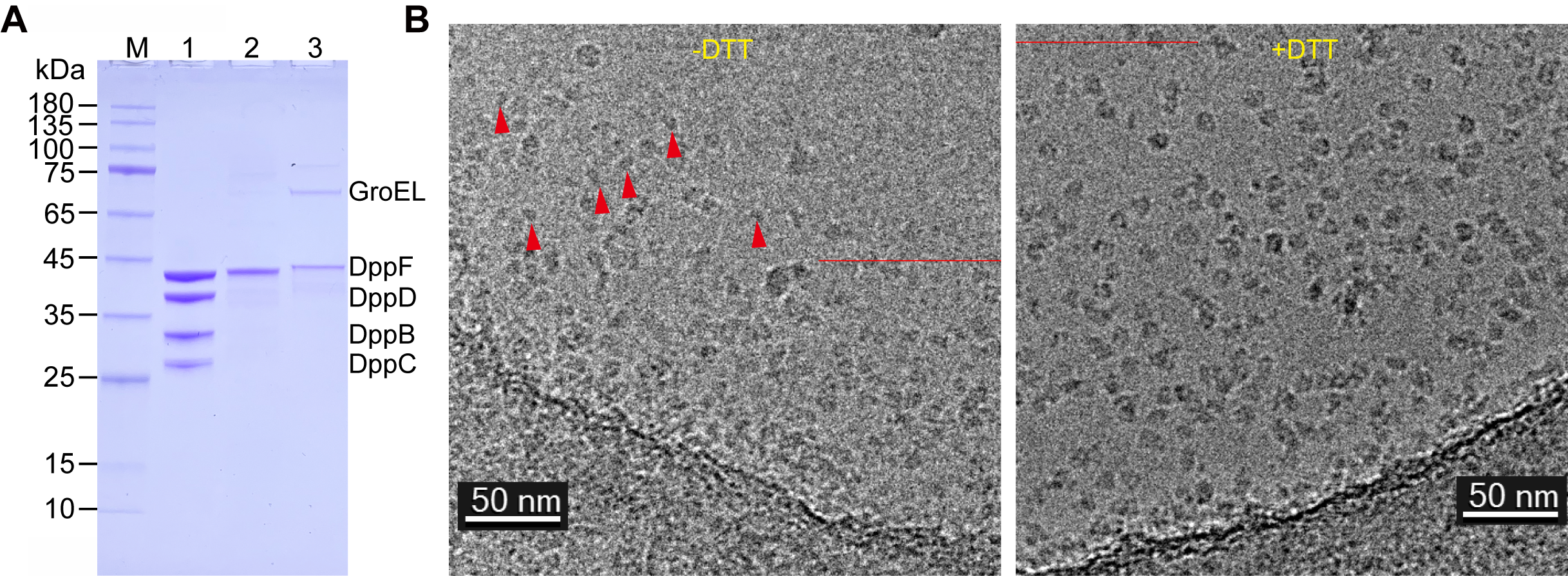

Supplement: S5 Fig — (A) 12% SDS-PAGE analysis of the purified DppBCDF variants. Lane 1, affinity-purified wild-type DppBCDF; lane 2, affinity-purified DppBCDC284S+C290S+C297S+C315SF; lane 3, affinity-purified DppBCDFC293S+C299S+C306S+C323S. Cys-to-Ser mutation in either DppD or DppF resulted in no pull-down of the DppBCDF complex. (B) The purified DppBCDF complex exhibited highly morphological difference in EM micrographs in the absence of DTT. Reducing condition is critical for Cys residues in both DppD and DppF chelating the [4Fe-4S] cluster and stabilizing the overall structure of DppBCDF. The data underlying this figure can be found in S1 Raw Images. (TIF) [file pbio.3003026.s005.tif]

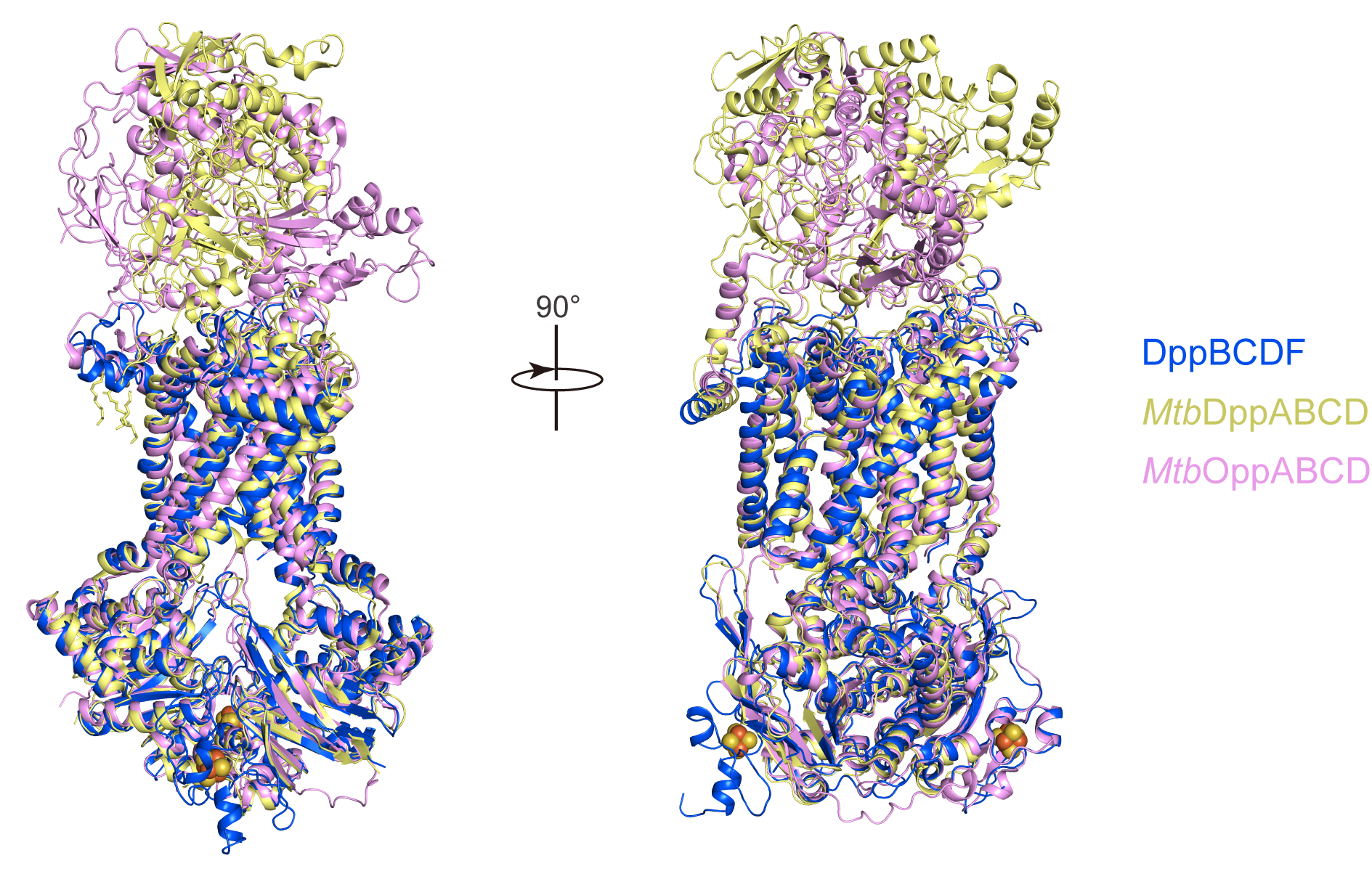

Supplement: S6 Fig — Cartoon representation of DppBCDF and MtbDppABCD/MtbOppABCD structures from two viewpoints. (TIF) [file pbio.3003026.s006.tif]

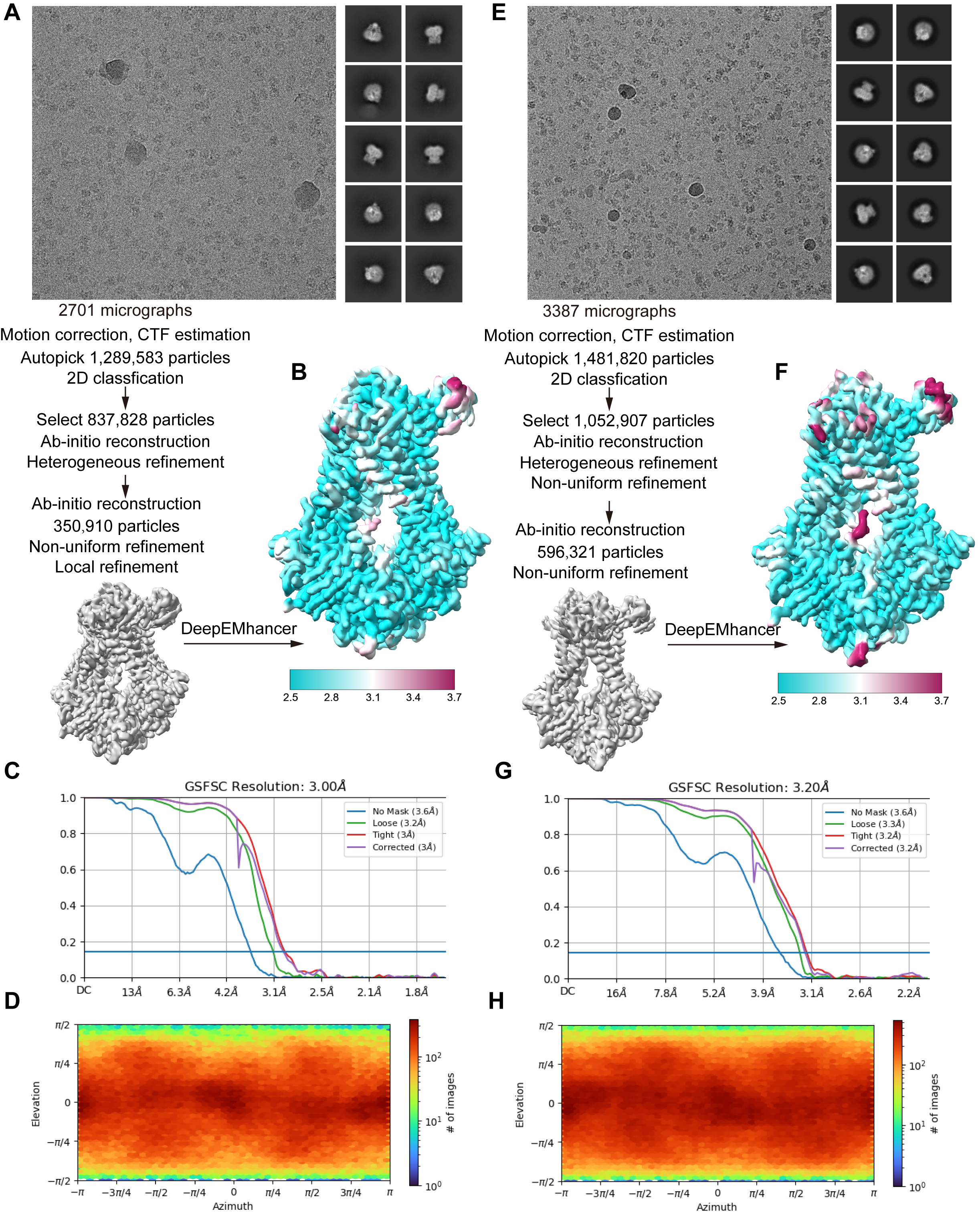

Supplement: S7 Fig — (A) Representative raw micrograph and 2D classification of the ATPγS-DppBCDF complex and schematic representation of the processing workflow. (B) Final deepEMhancer-postprocessed map colored according to the local resolution estimation in cryoSPARC. (C) Fourier shell correlation (FSC) curves of ATPγS-DppBCDF. (D) Angular distributions of ATPγS-DppBCDF. (E) Representative raw micrograph of the AMPPNP-DppBCDF complex and 2D classification and schematic representation of the processing workflow. (F) Final deepEMhancer-postprocessed map colored according to the local resolution estimation in cryoSPARC. (G) FSC curves of AMPPNP-DppBCDF. (H) Angular distributions of AMPPNP-DppBCDF. (TIF) [file pbio.3003026.s007.tif]

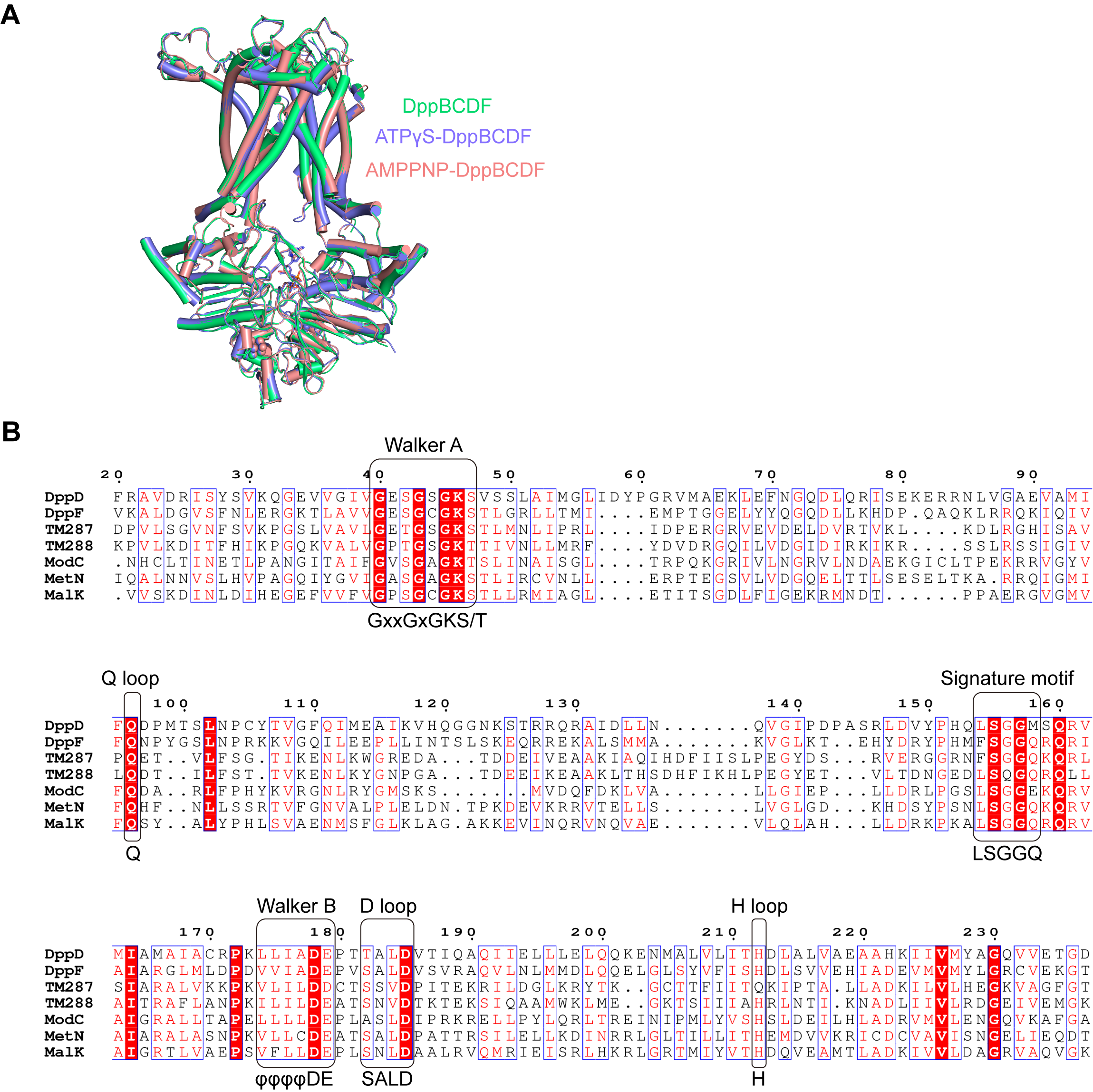

Supplement: S8 Fig — (A) Structural overlay of DppBCDF (lime), ATPγS-DppBCDF (slate blue) and AMPPNP-DppBCDF (salmon) showing no conformation differences among three structures. (B) Sequence alignment of DppD and DppF with ATPase sequences containing consensus sites (TM287, ModC, MetN, MalK) and degenerate site (TM288). (TIF) [file pbio.3003026.s008.tif]

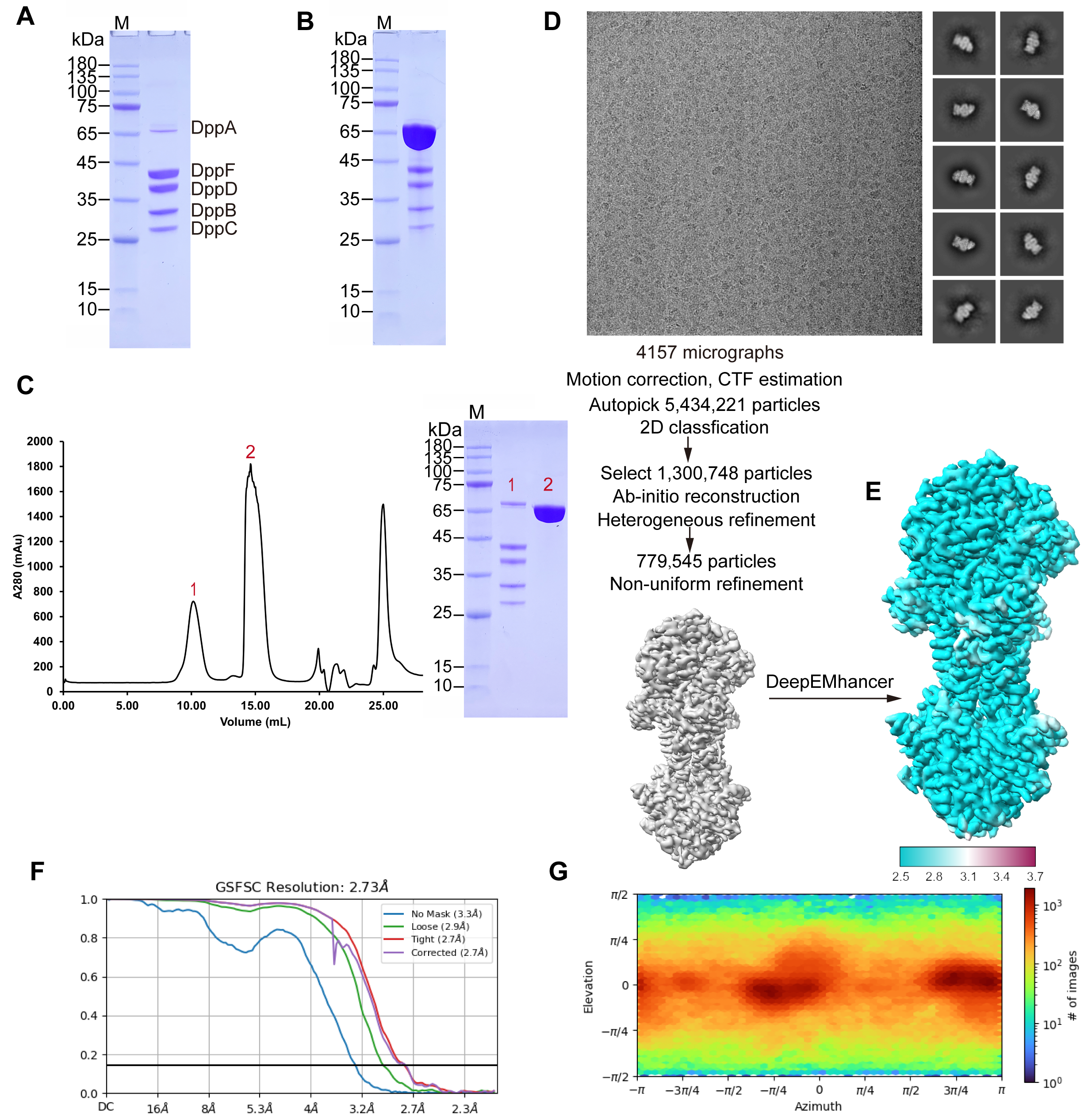

Supplement: S9 Fig — (A) The affinity-purified DppBCDE179QFE187Q complex pull-downed slight amount of endogenous DppA. (B) Co-expression and His-tag affinity-purification of DppABCDE179QFE187Q via DppA-His. (C) Gel filtration chromatographic profile of the affinity-purified DppABCDE179QFE187Q. (D–G) Schematic representation of the processing workflow of ATPγS-DppABCDF complex. Representative raw micrograph and 2D classes (D), and final deepEMhancer-postprocessed map colored according to the local resolution estimation in cryoSPARC (E). FSC curve (F) and angular distributions (G) used for the final reconstruction of particles generated by cryoSPARC. The data underlying this figure can be found in S1 Raw Images. (TIF) [file pbio.3003026.s009.tif]

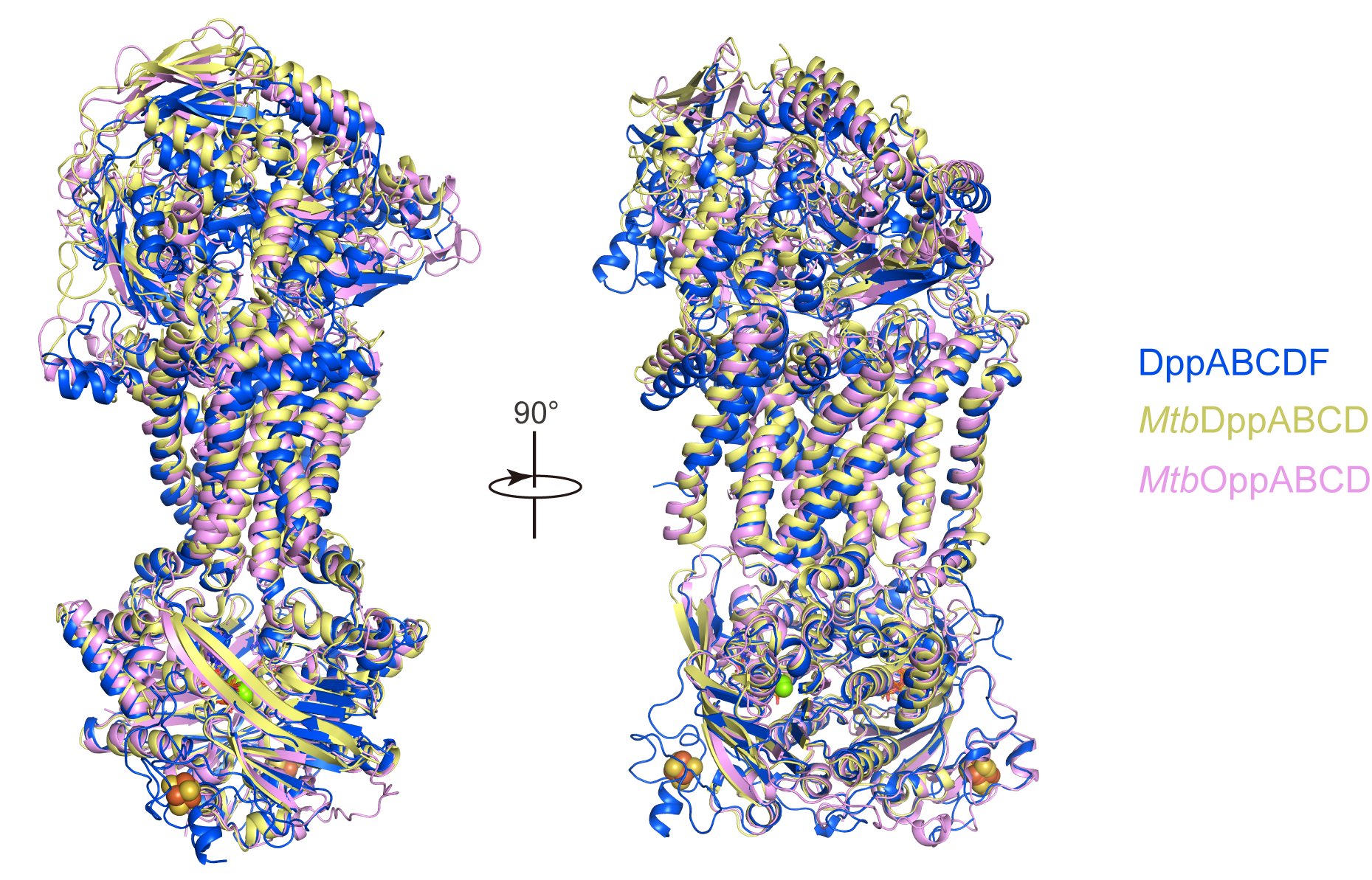

Supplement: S10 Fig — Cartoon representation of DppABCDF and MtbDppABCD/MtbOppABCD structures from two viewpoints. (TIF) [file pbio.3003026.s010.tif]

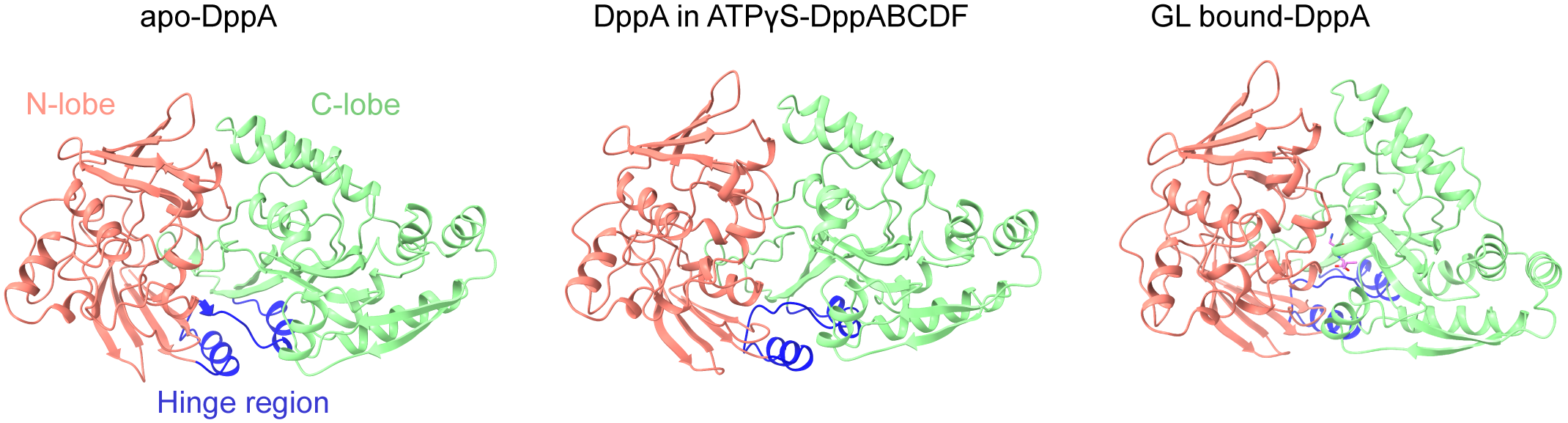

Supplement: S11 Fig — Apo-DppA adopts an open and substrate accessible conformation (left); DppA adopts a semi-open conformation in ATPγS-DppABCDF (middle); DppA adopts a clamped conformation in GL-bound state (right). (TIF) [file pbio.3003026.s011.tif]

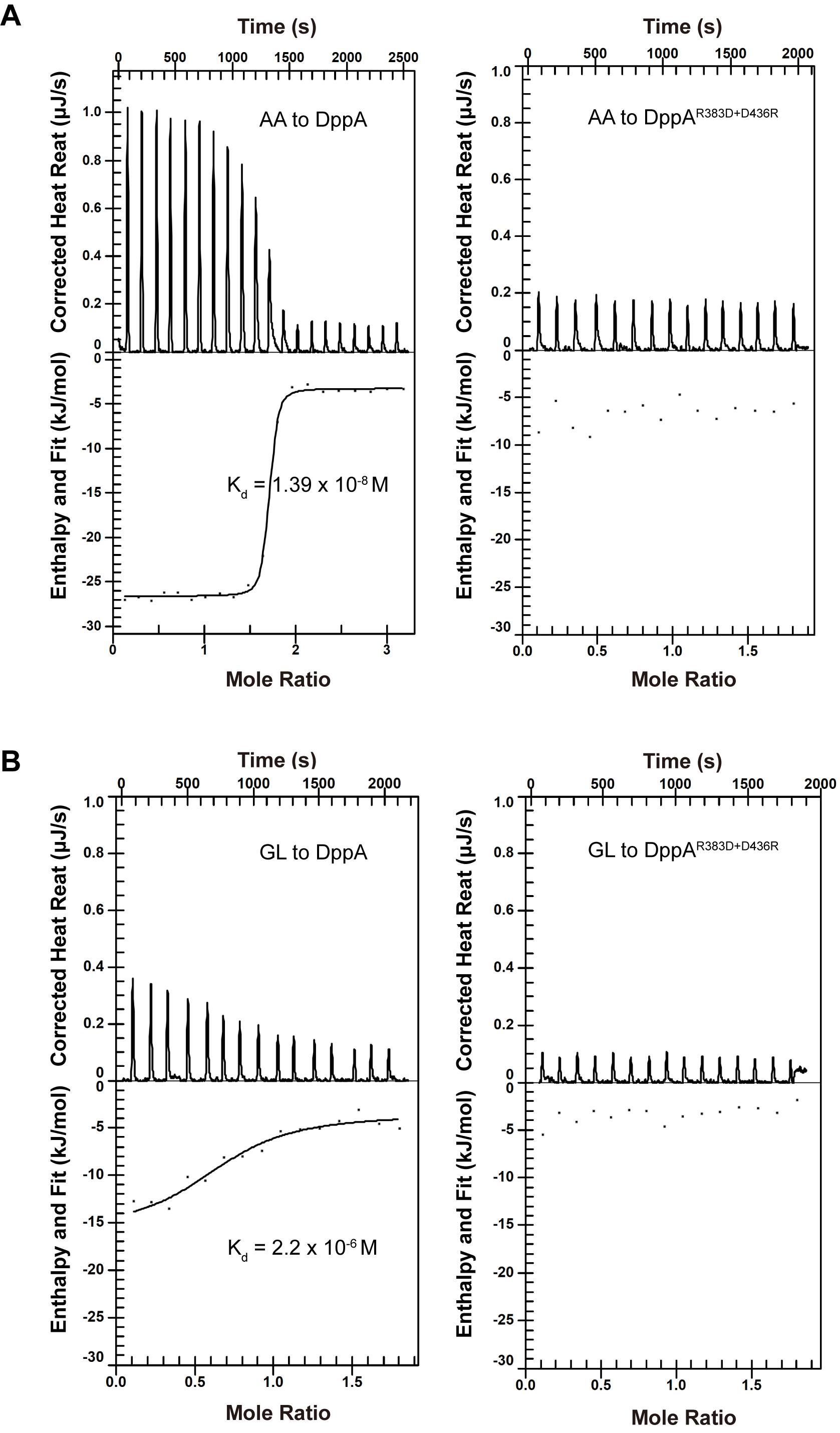

Supplement: S12 Fig — (A) ITC measurements of the affinity between wild-type DppA (or DppAR383D+D436R) and dipeptides AA. The affinity between wild-type DppA and AA is approximately 13.9 nM (left), while DppAR383D+D436R lost binding affinity to AA (right). (B) ITC measurements of the affinity between wild-type DppA (or DppAR383D+D436R) and dipeptides GL. The affinity between wild-type DppA and GL is approximately 2.2 μM (left), while DppAR383D+D436R lost binding affinity to dipeptide GL (right). The data underlying this figure can be found in S1 Data. (TIF) [file pbio.3003026.s012.tif]
