# Supplementary material for: Structural characterization of the ABC transporter DppABCDF in Escherichia coli reveals insights into dipeptide acquisition
Source: PLoS Biol. 2025 Mar 7;23(3):e3003026. doi: 10.1371/journal.pbio.3003026 (PMC12136057; doi:10.1371/journal.pbio.3003026)
Supplement: S1 Table — (DOCX) [file pbio.3003026.s013.docx]

**S1 Table. Cryo-EM data collection, refinement and validation statistics**

|  | DppBCDF  (EMD-39737)  (PDB: 8Z1V) | ATPγS-DppBCDF  (EMD-39738)  (PDB: 8Z1W) | AMPPNP-DppBCDF  (EMD-39739)  (PDB: 8Z1X) | ATPγS-DppABCDF  (EMD-39740)  (PDB: 8Z1Y) |
| --- | --- | --- | --- | --- |
| **Data collection and processing** |  |  |  |  |
| Magnification | 130,000× | 165,000× | 130,000× | 130,000× |
| Voltage (kV) | 300 | 300 | 300 | 300 |
| Electron exposure (e^-^/Å^2^) | 60 | 45 | 60 | 40 |
| Defocus range (μm) | -1.5~-2.5 | -1.2~-1.5 | -1.2~-2.2 | -1.2~-2.0 |
| Pixel size (Å) | 1.04 | 0.82 | 1.04 | 1.04 |
| Symmetry imposed | C1 | C1 | C1 | C1 |
| Initial particle images (no.) | 1,936,296 | 1,289,583 | 1,481,820 | 5,434,221 |
| Final particle images (no.) | 411,381 | 350,910 | 596,321 | 611,847 |
| Map resolution (Å) | 3.16 | 3.0 | 3.2 | 2.73 |
| FSC threshold | 0.143 | 0.143 | 0.143 | 0.143 |
| **Refinement** |  |  |  |  |
| Model resolution (Å) | 3.1/3.3 | 3.0/3.1 | 3.2/3.4 | 2.7/3.0 |
| FSC threshold | 0.143/0.5 | 0.143/0.5 | 0.143/0.5 | 0.143/0.5 |
| Map sharpening B factor (Å^2^) | -154.9 | -97.9 | -105.8 | -97.6 |
| Model composition |  |  |  |  |
| Non-hydrogen atoms | 9,562 | 9,622 | 9,649 | 13,897 |
| Protein residuces | 1,234 | 1,237 | 1,240 | 1,773 |
| Ligands | SF4: 2 | SF4: 2/AGS: 1 | SF4: 2/ANP: 1 | SF4: 2/AGS: 2 |
| B factor (Å^2^) |  |  |  |  |
| Protein | 151.8 | 130.2 | 126.8 | 113.61 |
| Ligand | 168.4 | 152 | 162.3 | 110.3 |
| R.m.s.deviations |  |  |  |  |
| Bond lengths (Å) | 0.025 | 0.018 | 0.019 | 0.015 |
| Bond angles (°) | 2.298 | 2.083 | 2.126 | 2.077 |
| Validation |  |  |  |  |
| Molprobity score | 0.68 | 0.52 | 0.61 | 0.61 |
| Clashscore | 0.52 | 0.05 | 0.31 | 0.25 |
| Poor rotamers (%) | 0 | 0.1 | 0 | 0.07 |
| Ramachandran plot |  |  |  |  |
| Favored (%) | 99.35 | 98.7 | 98.78 | 97.96 |
| Allowed (%) | 0.65 | 1.3 | 1.22 | 2.04 |
| Disallowed (%) | 0 | 0 | 0 | 0 |
