# Supplementary material for: Structural characterization of the ABC transporter DppABCDF in Escherichia coli reveals insights into dipeptide acquisition
Source: PLoS Biol. 2025 Mar 7;23(3):e3003026. doi: 10.1371/journal.pbio.3003026 (PMC12136057; doi:10.1371/journal.pbio.3003026)

Fig 1G Raw image of the BN-PAGE gel.

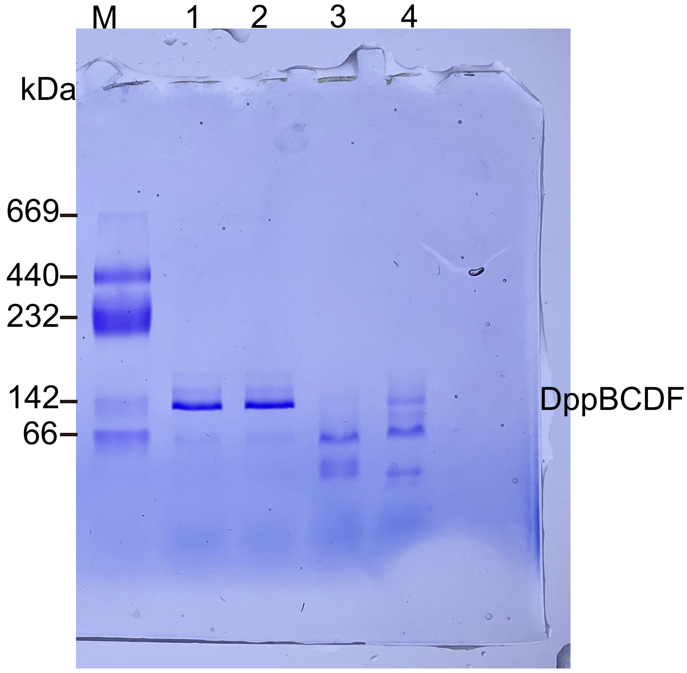

Fig S2B Raw image of the SDS-PAGE gel.

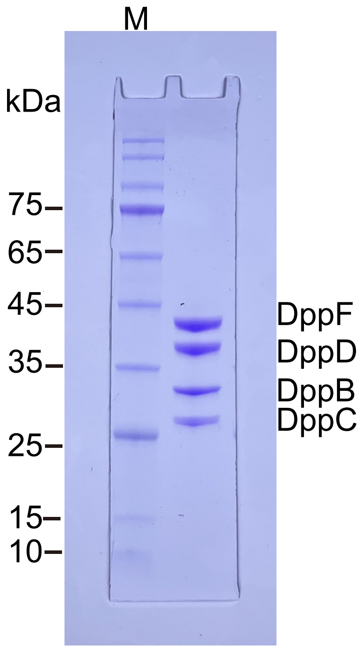

Fig S5A Raw image of the SDS-PAGE gel.

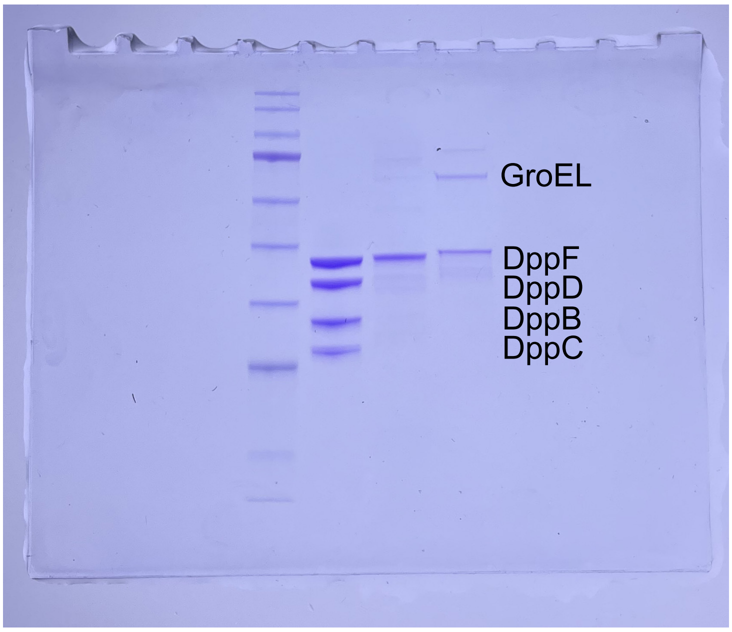

Fig S9A Raw image of the SDS-PAGE gel.

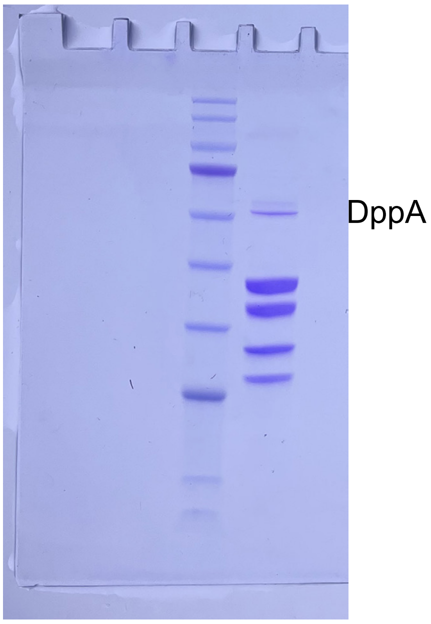

Fig S9B Raw image of the SDS-PAGE gel.

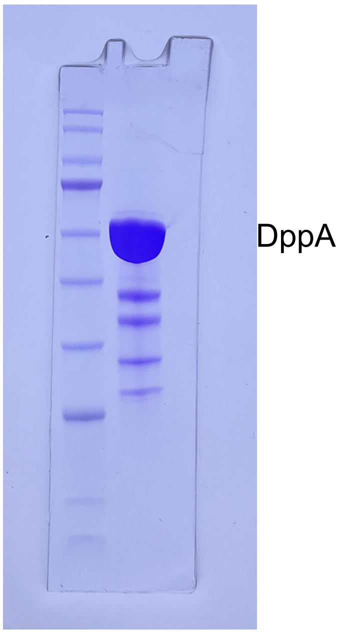

Fig S9C Raw image of the SDS-PAGE gel.

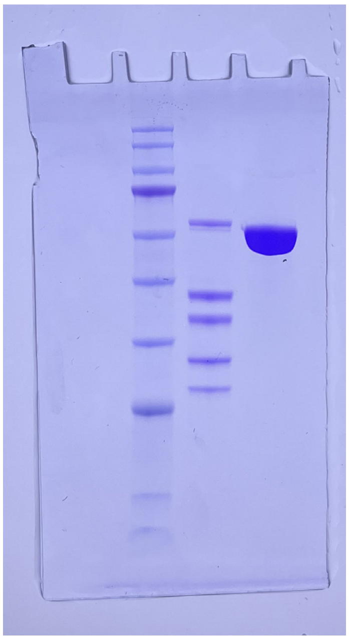

Fig 4C Raw images of complementation assays.

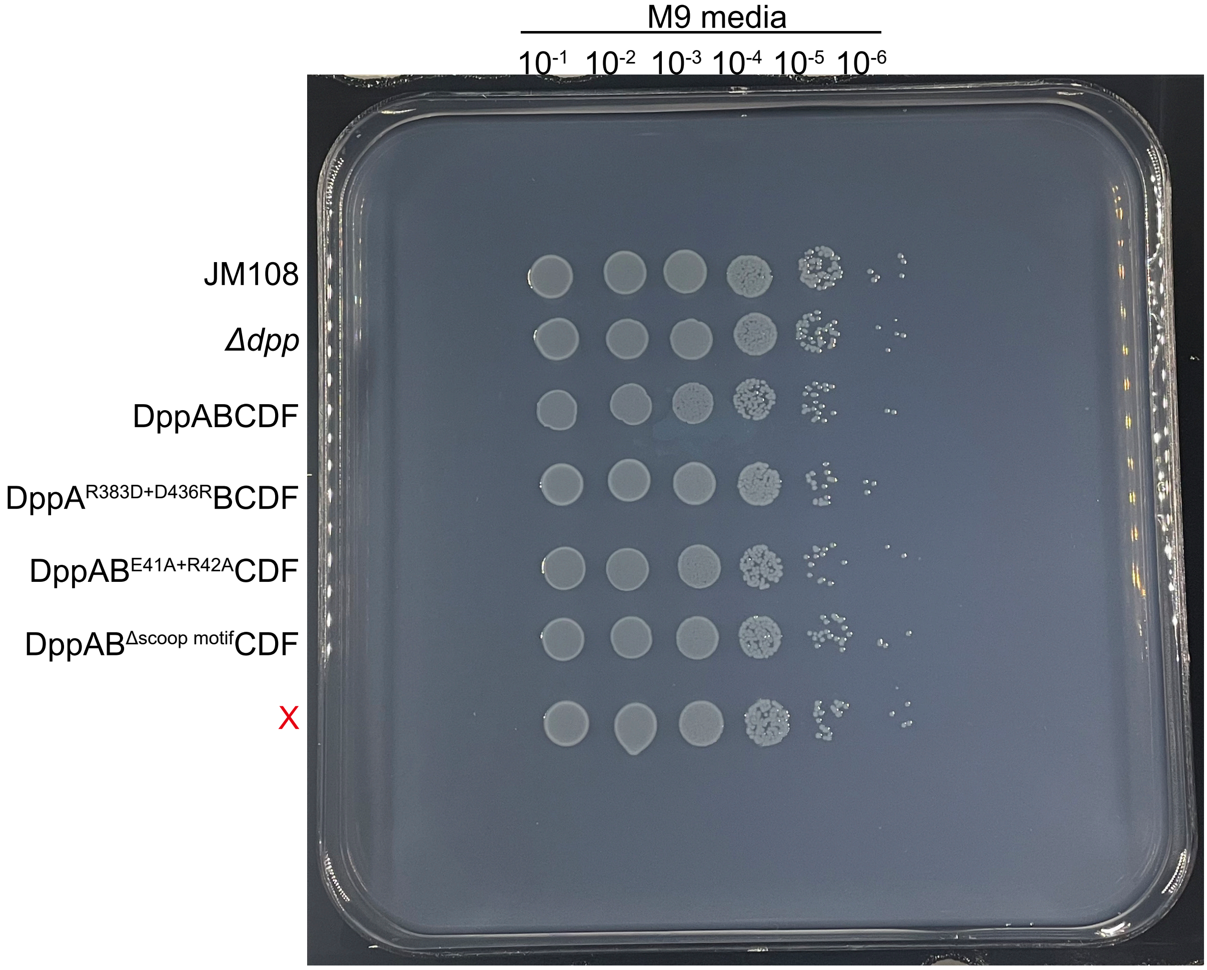

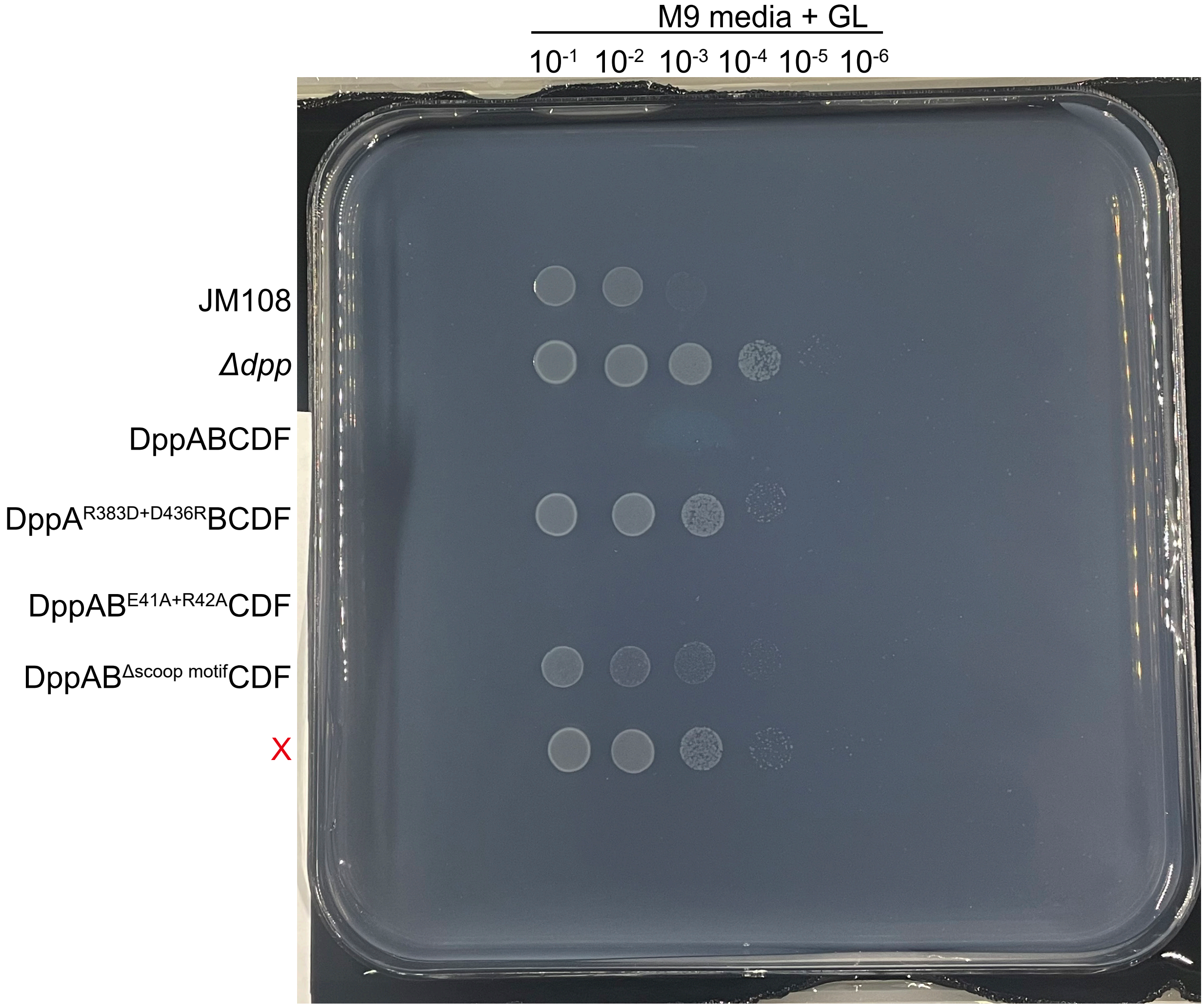

Supplement: S1 Raw Images — The corresponding figure numbers are indicated above each image. (PDF) [file pbio.3003026.s015.pdf]
